# JRAB/MICAL-L2 undergoes liquid–liquid phase separation to form tubular recycling endosomes

**DOI:** 10.1038/s42003-021-02080-7

**Published:** 2021-05-11

**Authors:** Ayuko Sakane, Taka-aki Yano, Takayuki Uchihashi, Kazuki Horikawa, Yusuke Hara, Issei Imoto, Shusaku Kurisu, Hiroshi Yamada, Kohji Takei, Takuya Sasaki

**Affiliations:** 1 Department of Biochemistry, Tokushima University Graduate School of Medical Sciences, Tokushima, Japan; 2 Department of Interdisciplinary Researches for Medicine and Photonics, Institute of Post-LED Photonics, Tokushima, Japan; 3 Department of Post-LED Photonics Research, Institute of Post-LED Photonics, Tokushima, Japan; 4 Department of Physics, Nagoya University, Nagoya, Japan; 5 Exploratory Research Center on Life and Living Systems, National Institutes of Natural Sciences, Okazaki, Aichi Japan; 6 Department of Optical Imaging, Advanced Research Promotion Center, Tokushima University, Tokushima, Japan; 7 Division of Molecular Genetics, Aichi Cancer Center Research Institute, Nagoya, Japan; 8 Department of Cancer Genetics, Nagoya University Graduate School of Medicine, Nagoya, Japan; 9 Department of Cell Biology, Tokushima University Graduate School of Medical Sciences, Tokushima, Japan; 10 Department of Neuroscience, Okayama University Graduate School of Medicine, Dentistry and Pharmaceutical Sciences, Okayama, Japan

**Keywords:** Endosomes, Small GTPases

## Abstract

Elongated tubular endosomes play essential roles in diverse cellular functions. Multiple molecules have been implicated in tubulation of recycling endosomes, but the mechanism of endosomal tubule biogenesis has remained unclear. In this study, we found that JRAB/MICAL-L2 induces endosomal tubulation via activated Rab8A. In association with Rab8A, JRAB/MICAL-L2 adopts its closed form, which functions in the tubulation of recycling endosomes. Moreover, JRAB/MICAL-L2 induces liquid–liquid phase separation, initiating the formation of tubular recycling endosomes upon overexpression. Between its N-terminal and C-terminal globular domains, JRAB/MICAL-L2 contains an intrinsically disordered region, which contributes to the formation of JRAB/MICAL-L2 condensates. Based on our findings, we propose that JRAB/MICAL-L2 plays two sequential roles in the biogenesis of tubular recycling endosomes: first, JRAB/MICAL-L2 organizes phase separation, and then the closed form of JRAB/MICAL-L2 formed by interaction with Rab8A promotes endosomal tubulation.

## Abstract (web summary)

Sakane et al. demonstrate that JRAB/MICAL-L2, an effector protein of Rab8 and Rab13, induces endosomal tubulation in HeLa cells depending on its closed conformation caused by an activated Rab8A. JRAB/MICAL-L2 undergoes liquid-liquid phase separation when overexpressed, which precedes its interaction with Rab8A, eventually leading to tubulation.

## Introduction

During fundamental cellular processes such as cell adhesion, migration, cell polarity, and signal transduction, the endocytic recycling pathways have a pivotal role in organizing the surface area and composition of the plasma membrane^1^. Various cell-surface proteins, including transmembrane receptors and lipids, are internalized and return to the plasma membrane via endocytic recycling. There are two different kinds of endocytic recycling pathways: fast recycling via a direct route from early endosomes, and slow recycling via an indirect route through the endocytic recycling compartment (ERC). In the latter type of recycling, dynamics of tubular endosomes have crucial roles in more efficient and reliable transport of cargo to the cell surface. First, cargos on the tips of elongated tubular endosome approach their destination, and are then separated from the tube as vesicles. Thus, cargos may travel safely over a long distance via a specific structure, i.e., the elongated tubular endosome. Several key regulatory proteins involved in the formation and maintenance of endosomal tubules have been discovered, including the small GTPases Rab8, Rab10, and Rab35, Arf6, Eps15 homology domain protein 1 (EHD-1), syndapin2 (an F-BAR protein), and MICAL-L1, which acts as a hub connecting the other factors^2–5^. In addition, recent studies showed that MICAL-L1 and syndapin2 localize on tubular recycling endosomes via phosphatidic acid (PA), leading to the initiation of endosomal tubulation^6^. However, the mechanism of endosomal tubule biogenesis has remained unclear.

MICAL-L1 belongs to the MICAL family, which has four other members in mammals: MICAL-1, MICAL-2, MICAL-3, and JRAB/MICAL-L2^7^. MICAL proteins are large, multidomain, cytosolic proteins expressed in specific neuronal and non-neuronal cells both during development and in adulthood. They contain calponin homology (CH), LIM, and coiled-coil (CC) domains, and MICAL-1, MICAL-2, and MICAL-3 also possess a flavin adenine dinucleotide-binding monooxygenase domain. In a previous study, we identified JRAB/MICAL-L2 as an effector protein for Rab8A and Rab13, as well as MICAL-L1^8^.

Small GTPases of the Rab family are involved in the regulation of membrane trafficking^9–14^. In mammalian cells, the Rab family consists of more than 70 members. Each Rab protein localizes on a distinct membrane organelle or plasma membrane, and then regulates the transport of proteins or lipids there. Each Rab protein exchanges between its GTP-bound active form and GDP-bound inactive form. GTP-bound Rab interacts with specific proteins, called effector proteins, and the Rab–effector protein complex provides each Rab with a specific function^15^. Thus, when determining the cellular function of a Rab protein, we must first identify its effector protein.

JRAB/MICAL-L2–Rab8A localizes in a perinuclear compartment (probably the ERC) and regulates the recycling of E-cadherin, whereas JRAB/MICAL-L2–Rab13 localizes underneath the plasma membrane and regulates the formation of tight junctions via the recycling of claudins and occludin^8,16^. We also demonstrated that JRAB/MICAL-L2 undergoes a conformational change between its closed and open forms depending on the association with Rab13, resulting in the spatiotemporal regulation of actin cytoskeleton during collective cell migration^17^. On the other hand, the effect of Rab8A on the conformational change of JRAB/MICAL-L2 remains unknown. MICAL-L1 is also an effector protein of Rab8A, and generates tubular endosomes accompanied by recruitment of Rab8A^18^. However, deletion of Rab8A has no effect on the tubular endosomes generated by MICAL-L1. In this study, we propose a new model in which JRAB/MICAL-L2 adopts its closed form through association with Rab8A, and that the JRAB/MICAL-L2–Rab8A complex is involved in endosomal tubulation. JRAB/MICAL-L2 has an intrinsically disordered region (IDR) between its N-terminal LIM domain and C-terminal CC domain. Recent work revealed the significance of IDR in liquid–liquid phase separation (LLPS)^19,20^. LLPS is initiated from liquid-like condensates of many biomolecules, including RNA and protein, and organizes diverse biological processes^21^. Here, we also show that the IDR-containing protein JRAB/MICAL-L2 forms phase-separated droplets along with Rab8A, which subsequently serve as sites of tubular endosome initiation. These results provide new insight into the biological mechanism underlying the tubulation of endosomes.

## Results

### JRAB/MICAL-L2 adopts its closed form upon interaction with Rab8A

Based on their amino acid sequences, Rab8A and Rab13 belong to the same subfamily of Rab GTPases^22,23^. In their GTP-bound active form, both proteins bind to the JRAB/MICAL-L2, indicating that Rab8A and Rab13 share JRAB/MICAL-L2 as a common effector protein^8^. However, the JRAB/MICAL-L2–Rab8A and JRAB/MICAL-L2–Rab13 complexes localize in completely different parts of the cells: the former at perinuclear regions and the latter at the plasma membrane^8^. In a recent study, we showed that the intramolecular interaction between JRAB-N and JRAB-C is competitively disrupted by binding of Rab13 to JRAB-C; i.e., interaction with Rab13 causes JRAB/MICAL-L2 to change its conformation from closed to open^17^. In this study, we examined the conformation of JRAB/MICAL-L2 under the influence of another Rab, Rab8A, reasoning that the result would provide clues about the mechanism by which Rab8A and Rab13 fulfill different functions at different places via their common effector protein.

**Fig. 1:**
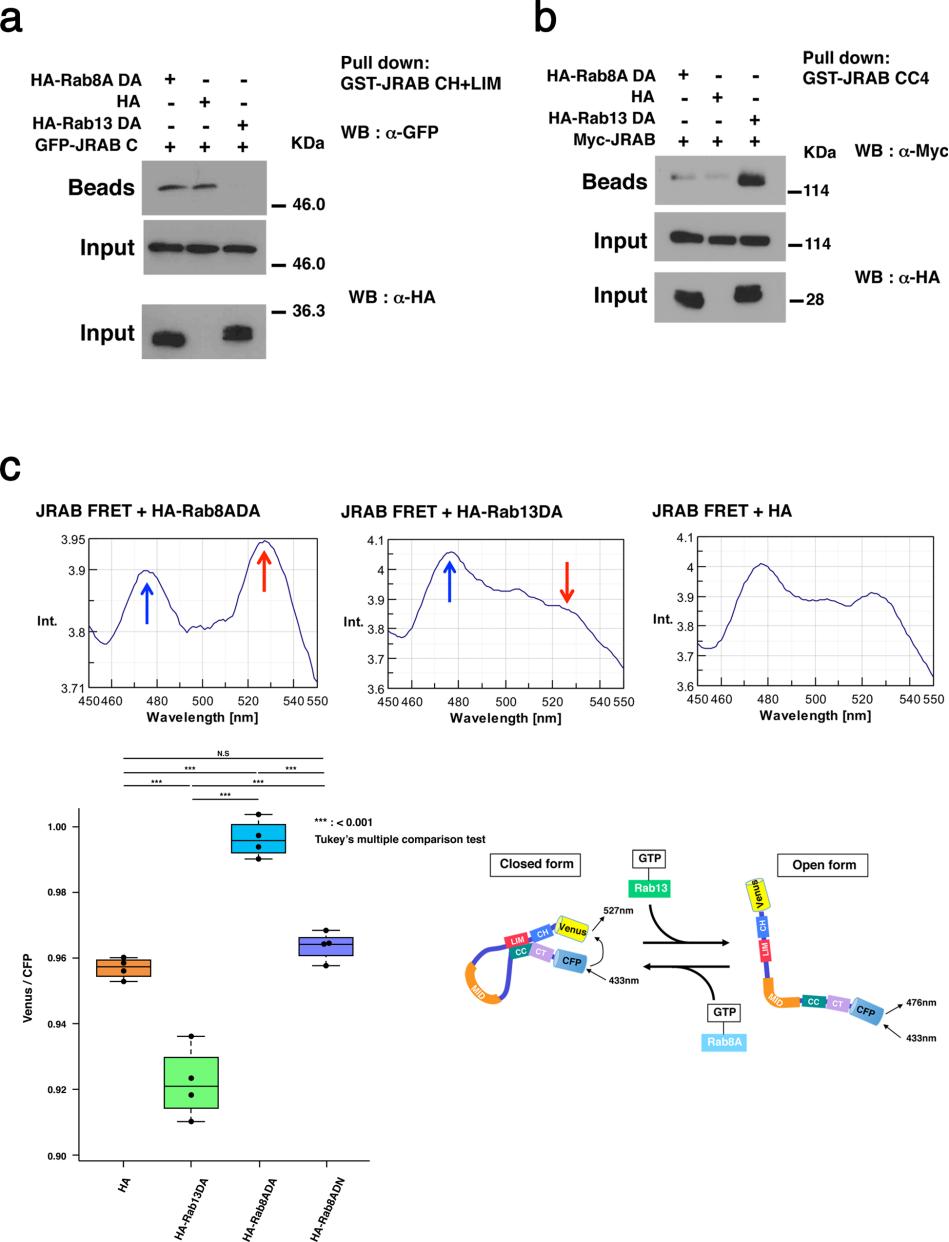
JRAB/MICAL-L2 adopts its closed form upon interacting with Rab8A. **a** Lysates of HEK293 cells co-expressing the GFP-tagged JRAB C-terminal region with HA-Rab13DA, HA-Rab8ADA, or HA were subjected to pulldown assays using GST-JRAB-CH+LIM. Pulled-down GFP-JRAB-C (Beads) was detected by western blotting (WB) with anti-GFP antibody. Total cell lysates (Input) were also analyzed with anti-GFP or anti-HA antibody. **b** HEK293 cell lysates containing Myc-JRAB with HA-Rab13DA, HA-Rab8ADA, or HA were subjected to pulldown assays using GST-JRAB-CC4 (aa 840–930), which binds to JRAB-CH+LIM, but not to either Rab8ADA or Rab13DA. Pulled-down Myc-JRAB (Beads) was detected by western blotting (WB) with anti-Myc antibody. Total cell lysates (Input) were also analyzed with anti-Myc or anti-HA antibody. **c** JRAB indicator was expressed in HEK293 cells with HA, HA-Rab8ADA, HA-Rab8ADN, or HA-Rab13DA, and the level of FRET in cell lysates was measured using a spectrofluorometer. (upper panels) Emission spectra of lysates from cells expressing JRAB indicator with HA, HA-Rab8ADA, or HA-Rab13DA were recorded at 1-nm intervals during excitation at 433 nm. (Lower left) Averaged emission ratios of Venus/CFP (*n* = 4) for HA, HA-Rab13DA, HA-Rab8ADA, and HA-Rab8ADN. Asterisks indicate statistical significance. Differences among groups were tested by ANOVA with Tukey’s post-hoc multiple comparison test. Differences were considered significant when *p* < 0.05. (Lower right, schematic) Proposed model for Rab8A- and Rab13-dependent conformational change of JRAB indicator. Experiments were repeated independently three (**a**, **b**) or four times (**c**) with similar results. Uncropped blots are shown in Supplementary Fig. 12 (**a**, **b**). Source data are available in Supplementary Data 1 (**c**).

First, we performed pulldown assays to determine whether Rab8A, like Rab13, is involved in disrupting the interaction between JRAB-N and JRAB-C. The presence of the HA-Rab8A dominant-active (DA) mutant (Q67L), which is defective in GTP hydrolysis, did not affect the interaction between GFP-JRAB-C and GST-JRAB-N (CH+LIM), whereas the HA-Rab13DA mutant (Q67L) inhibited the interaction, as we showed previously (Fig. 1a)^17^. These results indicate that the intramolecular interaction between JRAB-N and JRAB-C is not disrupted competitively by the binding of Rab8A to JRAB-C, in contrast to the case of Rab13. Next, to determine the effect of Rab8A on the full-length JRAB/MICAL-L2 structure, we performed pulldown assays using the GST-tagged JRAB-CC4 (amino acids [aa] 840–930), which binds to JRAB-CH+LIM, but neither Rab8ADA nor Rab13DA (Supplementary Fig. 1)^17^. In this assay, we can determine the proportion of JRAB/MICAL-L2 that is in the open form by the amount of full-length Myc-JRAB/MICAL-L2 pulled down. The result revealed that HA-Rab13DA, but not HA-Rab8ADA or HA alone, increases the proportion of Myc-JRAB/MICAL-L2 in the open form (Fig. 1b).

We verified the effect of Rab8A on the JRAB/MICAL-L2 structure using another approach. In this experiment, we introduced a JRAB indicator with an N-terminal fusion of Venus and a C-terminal fusion of CFP into HEK293 cells along with HA, HA-Rab8ADA, the HA-Rab8A dominant-negative mutant (DN; T22N), which has a lower affinity for GTP than GDP, or HA-Rab13DA, and then measured the level of fluorescence resonance energy transfer (FRET) in cell lysates using a spectrofluorometer^17^. As expected, we observed an activated Rab8A–dependent increase and activated Rab13-dependent decrease in FRET emission of the JRAB indicator (Fig. 1c). These data indicate that Rab8A maintains JRAB/MICAL-L2 in the closed form (Fig. 1c, schematic).

### JRAB/MICAL-L2 induces endosomal tubulation in association with Rab8A

Morphological dynamics of recycling endosomes have crucial roles in an efficient and reliable transport of cargos, such as proteins or lipids to cell surface^1,24,25^.

**Fig. 2:**
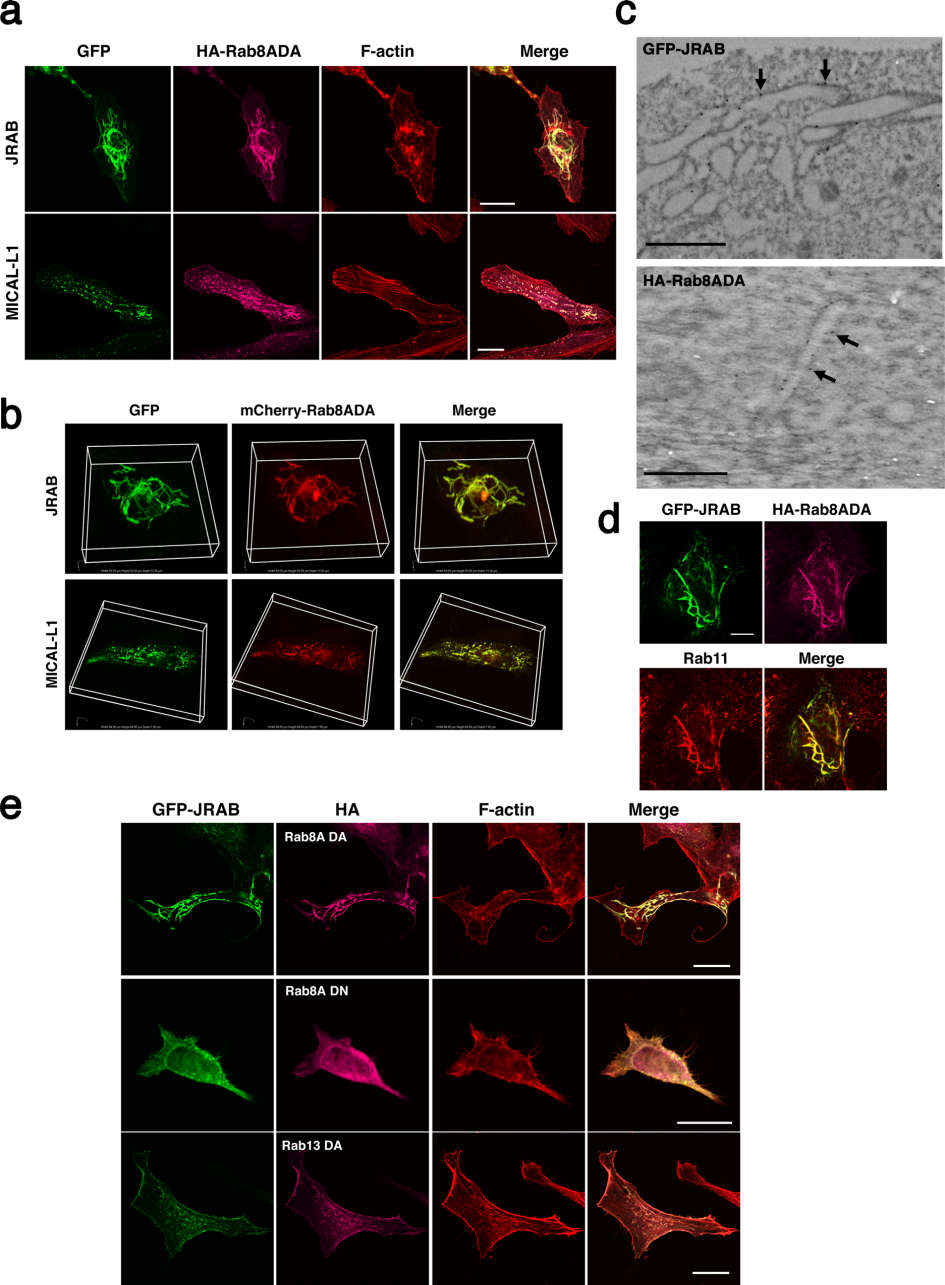
JRAB/MICAL-L2 induces endosomal tubulation along with Rab8A. **a** HeLa cells expressing HA-Rab8ADA with GFP-JRAB or GFP-MICAL-L1 were fixed and stained with anti-HA antibody, followed by rhodamine–phalloidin staining. Scale bar, 25 μm. **b** Representative 3D images of HeLa cells co-expressing mCherry-Rab8ADA and GFP-JRAB (upper panels: 53.03 μm × 53.03 μm × 13.20 μm) or GFP-MICAL-L1 (lower panels: 84.85 μm × 84.85 μm × 7.50 μm). **c** Immunoelectron microscopic images of HeLa cells co-expressing HA-Rab8ADA and GFP-JRAB. Immunoreactive GFP (JRAB) and HA (Rab8ADA) were distributed on the tubular membrane structures (arrows). Scale bar, 1 μm (upper), 500 nm (lower). **d** HeLa cells transfected with HA-Rab8ADA and GFP-JRAB were fixed and double-stained with anti-HA and anti-Rab11 antibodies. Scale bar, 10 μm. **e** HeLa cells expressing GFP-JRAB together with HA-Rab8ADA, HA-Rab8ADN, or HA-Rab13DA were fixed and stained with anti-HA antibody, followed by rhodamine–phalloidin staining. Scale bar, 25 μm. More than 40 transfected cells from four individual preparations were examined, and representative images are shown (**a**, **b**, **d**, **e**).

Activated Rab8A is recruited to tubular recycling endosomes via MICAL-L1, which is a main regulator of endosomal tubulation, as well as the EHD protein^18^. Both MICAL-L1 and JRAB/MICAL-L2 bind activated Rab8A^8^. To examine the involvement of the JRAB/MICAL-L2–Rab8A complex in the biogenesis of tubular recycling endosomes, we prepared HeLa cells expressing the recombinant proteins indicated in Fig. 2a. To detect overall morphology, the cells were stained for F-actin. Both GFP-JRAB/MICAL-L2 and GFP-MICAL-L1 were colocalized with HA-Rab8ADA on tubular structures. In cells expressing GFP-JRAB/MICAL-L2 and HA-Rab8ADA, tubular endosomes were longer (continuous) and much more complicated than in cells expressing GFP-MICAL-L1 and HA-Rab8ADA (Fig. 2a). These findings were supported by 3D images of HeLa cells expressing mCherry-Rab8ADA with GFP-JRAB/MICAL-L2 or GFP-MICAL-L1 (Fig. 2b). Images acquired by immunoelectron microscopy revealed the localization of GFP-JRAB/MICAL-L2 and HA-Rab8ADA on the surface of lipid bilayer membranes of tubular structures (Fig. 2c). The tubular structures induced by GFP-JRAB/MICAL-L2 and HA-Rab8ADA were coated with Rab11, which is a marker for recycling endosomes (Fig. 2d). Markers for mitochondria and the endoplasmic reticulum were not localized on tubules coated with GFP-JRAB/MICAL-L2 and HA-Rab8ADA (Supplementary Fig. 2). CD147, transferrin receptor, and MICAL-L1, three other markers for recycling endosomes, were partially colocalized with GFP-JRAB/MICAL-L2 and HA-Rab8ADA at tubular structures (Supplementary Fig. 2). MICAL-L1 forms tubular endosomes even in the absence of Rab8^18^. By contrast, GFP-JRAB/MICAL-L2–positive tubular endosomes were observed in the presence of HA-Rab8ADA, but not in the presence of HA-Rab8ADN (Fig. 2e). In addition, when GFP-JRAB/MICAL-L2 was co-expressed with HA-Rab13DA in the cells, the proteins mainly localized on the plasma membrane, and no GFP-JRAB/MICAL-L2-positive tubular endosomes were observed in the cells (Fig. 2e).

To confirm that JRAB/MICAL-L2 is associated with a membrane of tubular recycling endosomes via Rab8A, we performed in vitro experiments using liposomes. For this study, we generated new constructs, pcDNA4-His-GFP-JRAB/MICAL-L2 and pcDNA4-His-mCherry-Rab8ADA, and then purified each recombinant protein from lysates of HEK293 cells transfected with the corresponding construct. Liposomes were prepared as mixtures of charge-neutral phosphatidylcholine (PC) and acidic phosphatidylserine (PS) phospholipids at a molar ratio of 7:3 (Supplementary Fig. 3a). When His-GFP-JRAB/MICAL-L2 and His-mCherry-Rab8ADA were mixed and incubated with liposomes, both proteins localized on liposomes and induced small bud-like structures from the surfaces of existing large liposomes (arrows, Supplementary Fig. 3b). It should be noted that His-mCherry-Rab8ADA alone could bind to liposomes but did not induce their deformation, whereas His-GFP-JRAB/MICAL-L2 alone neither bound to nor deformed the liposomes (Supplementary Fig. 3c). These results suggested that Rab8A serves as a molecular scaffold to recruit JRAB/MICAL-L2 to the endosomal membrane, leading to the membrane deformation.

Taken together, JRAB/MICAL-L2 is involved in the elongation of tubular endosomes by a specific interaction with activated Rab8A, but not with activated Rab13. Moreover, the properties of the tube seem to be different from those of tubes generated by MICAL-L1: the tubular structures induced by JRAB/MICAL-L2–Rab8A complex are long and continuous, whereas those induced by MICAL-L1 are short and scattered throughout the cytoplasm.

### The closed form of JRAB/MICAL-L2 is required for endosomal tubulation

**Fig. 3:**
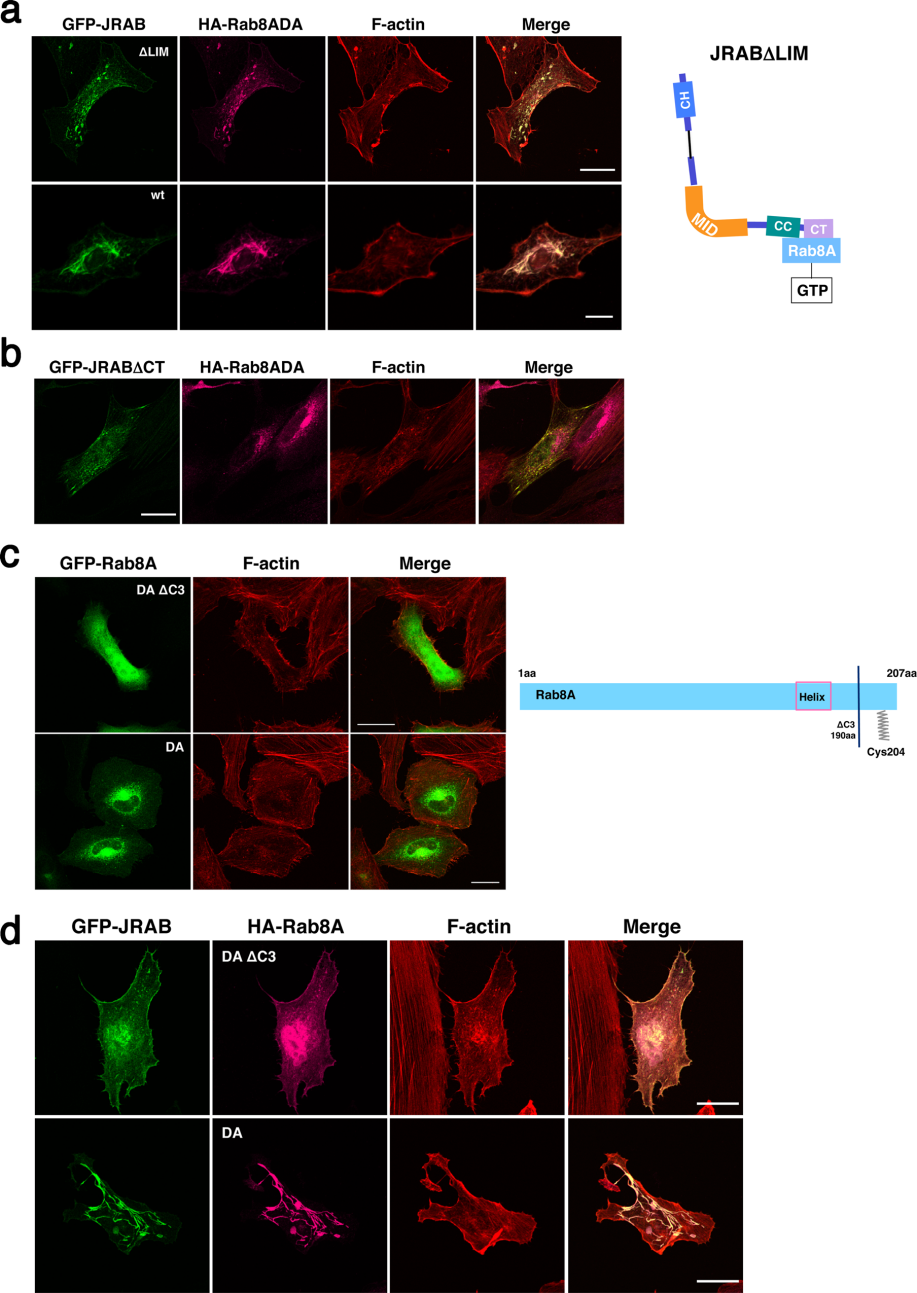
Association of the closed form JRAB/MICAL-L2 with Rab8A and C terminus of Rab8A Is necessary for endosomal tubulation. **a** HeLa cells expressing HA-Rab8ADA with GFP-JRABΔLIM (ΔLIM) or GFP-JRAB (wt) were fixed and stained with anti-HA antibody, followed by rhodamine–phalloidin staining. Scale bar, 25 μm (upper), 10 μm (lower). The schematic shows that JRABΔLIM associates with GTP-Rab8A (active form) in the open form. **b** HeLa cells transfected with GFP-JRABΔCT and HA-Rab8ADA were fixed and stained with anti-HA antibody, followed by rhodamine–phalloidin staining. Scale bars, 25 μm. **c** HeLa cells transfected with GFP-Rab8ADAΔC3 (upper panels) or GFP-Rab8ADA (lower panels) were fixed and stained with rhodamine–phalloidin. Scale bars, 25 μm. The schematic summarizes the sequence structure of the Rab8A truncated mutant. Cys204 represents the position of amino acid with a geranylgeranyl modification. **d** HeLa cells co-transfected with GFP-JRAB and HA-Rab8ADAΔC3 (upper panels) or HA-Rab8ADA (lower panels) were fixed and stained with anti-HA antibody, followed by rhodamine–phalloidin staining. Scale bar, 25 μm. More than 40 transfected cells from four individual preparations were examined, and representative images are shown (**a**–**d**).

We then examined whether the interaction between JRAB/MICAL-L2 and Rab8A is sufficient for the formation of tubular endosomes, regardless of the conformation of JRAB/MICAL-L2. We used the GFP-JRAB∆LIM deletion mutant, which lacks the LIM domain responsible for the intramolecular interaction^26^. GFP-JRAB∆LIM was pulled down by GST-JRAB-CH+LIM, indicating that JRABΔLIM is in the open form, whereas GFP-JRAB∆CT, a mutant that is maintained in the closed form, was not^26^. To assess the association of GFP-JRAB∆LIM with Rab8A, we performed immunoprecipitation assays. The results revealed that GFP-JRAB∆LIM binds to HA-Rab8ADA just like full-length GFP-JRAB (Supplementary Fig. 4a). Specifically, JRAB∆LIM associates with Rab8A, but the conformation is constitutively open (Fig. 3a, schematic). We then investigated the formation of tubular endosomes using GFP-JRAB∆LIM in HeLa cells. GFP-JRAB∆LIM did not induce the tubulation of endosomes even in the presence of HA-Rab8ADA, in contrast to full-length GFP-JRAB (Fig. 3a). Next, we examined the tubulation of endosomes in HeLa cells expressing GFP-JRAB∆CT; JRAB∆CT adopts the closed form, but does not contain the Rab8A-binding domain. In these cells, no tubular structure was observed in the presence or absence of HA-Rab8ADA (Fig. 3b and Supplementary Fig. 4b). Together, these observations suggest that the association of the closed form of JRAB/MICAL-L2 with Rab8A is required for endosome tubulation.

### The C terminus of Rab8A is necessary for tubulation of endosomes induced by JRAB/MICAL-L2

The C-terminal region of Rab8A receives a geranylgeranyl modification on cysteine 204 (Cys204) that is required for recruitment to the lipid bilayer membrane (recycling endosomes in the case of Rab8A)^27,28^. To investigate the importance of this lipid modification, we prepared a Rab8ADA mutant (Rab8ADA∆C3) that lacks the C-terminal region (aa 191–207), including Cys204 (Fig. 3c, schematic). Pulldown assays revealed that GFP-Rab8ADA∆C3 bound to GST-JRAB-C as strongly as GFP-Rab8ADA. As a negative control we used another mutant, Rab8ADA∆C1, which lacks the C-terminal region (aa 167–207), including helix α5, (Supplementary Fig. 4c). GFP-Rab8ADA∆C3 localized diffusely, rather than specifically on recycling endosomes (Fig. 3c). Moreover, GFP-JRAB/MICAL-L2 did not induce tubular endosomes in the presence of HA-Rab8ADA∆C3 (Fig. 3d). These results indicate that Rab8A connects JRAB/MICAL-L2 with the endosomal membrane via its C terminus (probably the lipid modification on Cys204), resulting in the elongation of the recycling endosome.

### The structure of JRAB/MICAL-L2 is determined by the difference between the C-terminal regions of Rab8A and Rab13

**Fig. 4:**
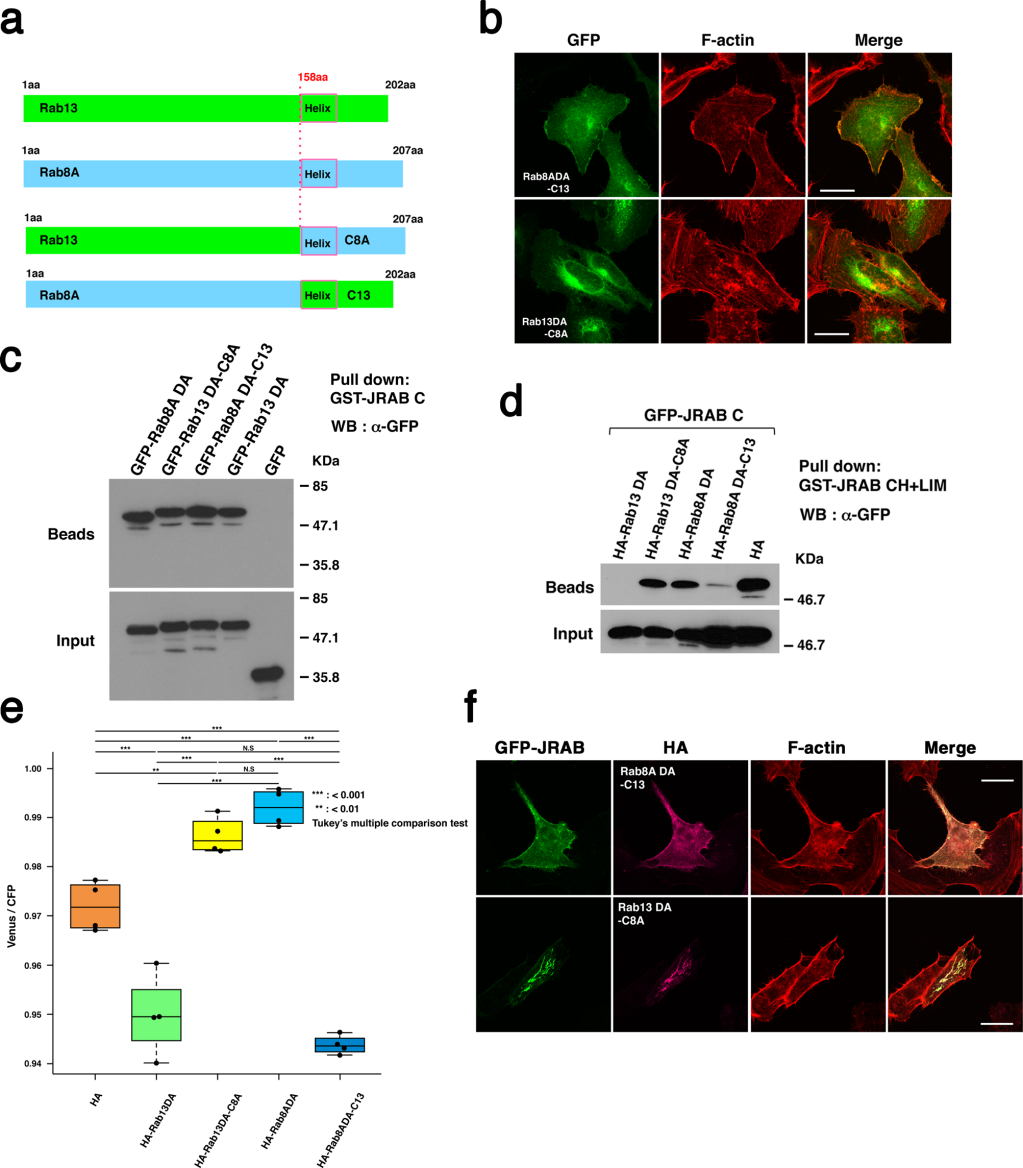
The structure of JRAB/MICAL-L2 is determined by the difference between the C-terminal regions of Rab8A and Rab13. **a** Schematic diagrams summarizing the construction of Rab8A, Rab13, and their chimeric variants, Rab8ADA-C13 and Rab13DA-C8A, in which the C-terminal regions (including the C-terminal variable regions) were reciprocally exchanged. **b** HeLa cells transfected with GFP-Rab8ADA-C13 (upper panels) or -Rab13DA-C8A (lower panels) were fixed and stained with rhodamine–phalloidin. Scale bars, 25 μm. **c** HEK293 cell lysates containing GFP-Rab13DA, -Rab8DA, or their chimeric variants were subjected to pulldown assays using GST-JRAB-C. Pulled-down protein (Beads) and the amount of expressed protein in total cell lysates (Input) were detected by western blotting (WB) with anti-GFP antibody. **d** Lysates of HEK293 cells co-expressing GFP-JRAB-C and HA-Rab13DA, HA-Rab13DA-C8A, HA-Rab8DA, or HA-Rab8DA-C13 were subjected to pulldown assays using GST-JRAB-CH+LIM. Pulled-down protein (Beads) and the amount of expressed protein in total cell lysates (Input) were detected by western blotting (WB) with anti-GFP antibody. **e** JRAB indicator was co-expressed in HEK293 cells along with HA-Rab13DA, HA-Rab13DA-C8A, HA-Rab8ADA, HA-Rab8A-C13, or HA, and the level of FRET in cell lysates was measured using a spectrofluorometer. Averaged emission ratios of Venus/CFP (*n* = 4) are plotted. Asterisks indicate statistical significance. Differences among groups were tested by ANOVA with Tukey’s post-hoc multiple comparison test. Differences were considered significant when *p* < 0.05. **f** HeLa cells co-expressing GFP-JRAB and HA-Rab8ADA-C13 or -Rab13DA-C8A were fixed and stained with anti-HA antibody, followed by rhodamine–phalloidin staining. Scale bar, 25 μm. More than 40 transfected cells from four individual preparations were examined, and representative images are shown (**b**, **f**). Experiments were repeated independently three (**c**, **d**) or four times (**e**) with similar results. Uncropped blots are shown in Supplementary Fig. 12 (**c**, **d**). Source data are available in Supplementary Data 1 (**e**).

Each Rab protein contains a variable region at its C terminus, which confers individual specificity of cellular localization^28–30^. Alignments of Rab13 and Rab8A proteins revealed low similarity in their C-terminal variable regions following helix α5 (aa 158–175). We postulated that the difference between the C-terminal variable regions of Rab8A and Rab13 determines not only the functional place but also the conformation of JRAB/MICAL-L2, leading to a specific function. To verify this assumption, we prepared the chimeric constructs Rab8ADA-C13 and Rab13DA-C8A by reciprocally exchanging the C-terminal region including the variable region: Rab8ADA-C13 consists of the N-terminal 157 residues of Rab8ADA fused to the C-terminal 46 residues of Rab13, and Rab13DA-C8A consists of the N-terminal 157 residues of Rab13DA fused to the C-terminal 51 residues of Rab8A (Fig. 4a, schematic). In HeLa cells, the localization of the chimeric proteins was governed by their C terminus; Rab8ADA-C13 localized at plasma membrane, whereas Rab13DA-C8A localized at perinuclear regions (Fig. 4b). It should be noted that Rab8ADA localized at perinuclear regions, whereas Rab13DA localized at plasma membrane, as previously shown^8^ (Supplementary Fig. 5). We also investigated whether both Rab8ADA-C13 and Rab13DA-C8A bound JRAB/MICAL-L2 like Rab8ADA and Rab13DA, respectively. In the pulldown assay, GFP-Rab8ADA-C13 and GFP-Rab13DA-C8A bound to GST-JRAB-C just as GFP-Rab8ADA and GFP-Rab13DA did (Fig. 4c). We then performed pulldown assays to examine the effect of exchanging the C-terminal regions of Rab8A and Rab13 on the interaction between JRAB-N and JRAB-C. HA-Rab8ADA-C13 recombinant protein inhibited the interaction between GFP-JRAB-C and GST-JRAB-N (CH+LIM), whereas HA-Rab13DA-C8A did not (Fig. 4d). This finding was consistent with the result from the experiment with the JRAB FRET indicator, which showed that Rab8ADA-C13 decreased FRET emission whereas Rab13DA-C8A increased it (Fig. 4e). Finally, we examined the effect of the chimeric proteins Rab8ADA-C13 and Rab13DA-C8A on the formation of tubular endosomes. When GFP-JRAB/MICAL-L2 was co-expressed with HA-Rab8ADA-C13 in HeLa cells, tubular endosome formation was not induced (Fig. 4f). By contrast, when GFP-JRAB/MICAL-L2 was co-expressed with HA-Rab13DA-C8A, elongated tubular endosomes were clearly detected (Fig. 4f). HA-Rab13DA-C8A could connect JRAB/MICAL-L2 with recycling endosomes, and this mutant fixed the structure of JRAB/MICAL-L2 in the closed form (Fig. 4d, e). Together, these results show that the specific C-terminal variable regions of Rab8A and Rab13 spatially regulate the conformation of JRAB/MICAL-L2, leading to multiple biological functions.

### The JRAB/MICAL-L2–Rab8A complex induces tubulation of recycling endosomes via liquid–liquid phase separation

**Fig. 5:**
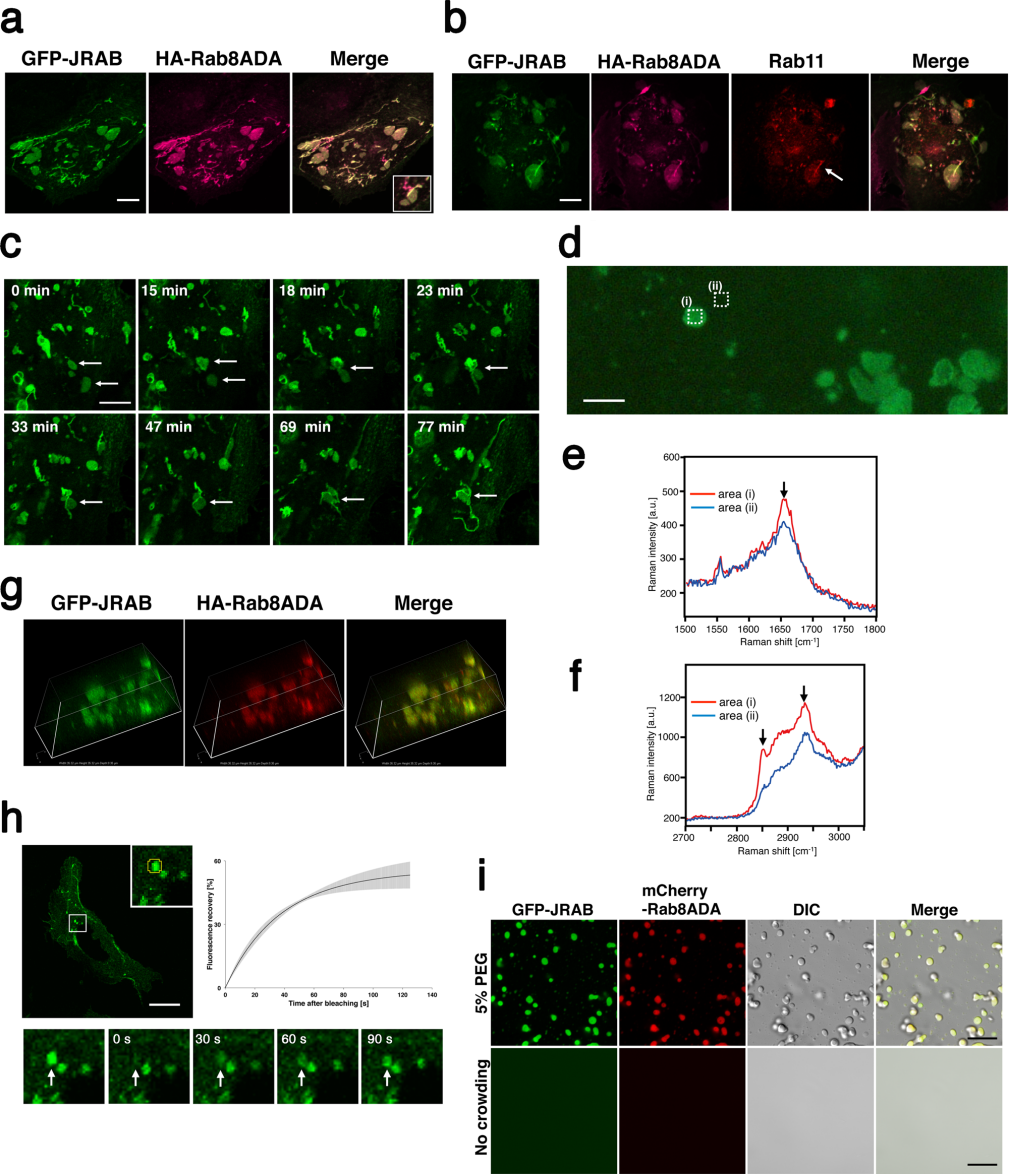
JRAB/MICAL-L2–Rab8A complex induces tubulation of recycling endosomes via liquid–liquid phase separation. **a** Twenty-four hours after transfection, HeLa cells co-expressing GFP-JRAB and HA-Rab8ADA were fixed and stained with anti-HA antibody. Scale bar, 10 μm. The inset on the right image shows an enlarged image, indicating that the tube budded from the pancake-like structure. **b** HeLa cells transfected with GFP-JRAB and HA-Rab8ADA were fixed and double-stained with anti-HA and anti-Rab11 antibodies. Arrow indicates localization of Rab11 on the tubular structure. Scale bar, 10 μm. **c** Representative time-lapse images of HeLa cells co-transfected with GFP-JRAB and HA-Rab8ADA. Each image was captured at the indicated time (min). After 18 min, two pancake-like droplets (arrows) fused with each other. Then, a tube emerged from the fused droplet (arrow) and elongated (see also Supplementary Movie 1). Scale bar, 10 μm. **d** Fluorescence image of PFA-fixed HeLa cells in PBS. Averaged Raman spectra in **e** low- and **f** high-frequency regions were measured inside (i) and outside (ii) the droplet indicated by the dotted areas in **d**. Scale bar, 5 μm. Averaged Raman intensity ratios between the interior and exterior of droplets containing GFP-JRAB and HA-Rab8ADA are shown in Supplementary Fig. 11. **g** Three-dimensional images of pancake-like structures. HeLa cells expressing GFP-JRAB and HA-Rab8ADA were fixed and stained with anti-HA antibody (35.32 μm × 35.32 μm × 9.35 μm). **h** (Upper panel) The square indicates a representative area containing the ROI for FRAP analysis. Scale bar, 25 μm. The inset is a magnified view of the boxed area. Yellow circle, ROI. The graph represents the recovery dynamics of the fluorescence signals, normalized against the signals before bleaching. Black is an average of fitted data for three independent experiments. Gray, s.d. (Lower panels) Representative FRAP images in the square of the upper panel. After bleaching, the GFP signal within the ROI of pancake-like structures recovered within a few minutes (see also Supplementary Movie 2); the arrow indicates the bleached area. **i** His-GFP-JRAB (green) and His-mCherry-Rab8ADA (red) exhibited liquid-like droplet formation in the presence of crowding agent (upper panels, 5% PEG; lower panels, no crowding agent). Scale bar, 10 μm. More than 40 transfected cells from four individual preparations were examined, and representative images are shown (**a**, **b**, **g**). Experiments were repeated independently three times, with similar results (**c**–**f**, **h**, **i**). Source data are available in Supplementary Data 1 (**h**).

We next examined how JRAB/MICAL-L2 and Rab8A form tubular recycling endosomes. For this purpose, we attempted to determine the structure of the pre-tubular endosome formed by JRAB/MICAL-L2–Rab8A complex. One day after co-transfection with GFP-JRAB/MICAL-L2 and HA-Rab8ADA, HeLa cells contained many pancake-like structures coated with GFP-JRAB/MICAL-L2 and HA-Rab8ADA (Fig. 5a). It should be noted that the marker for lipid droplets was not localized on the structure (Supplementary Fig. 6). In addition, some tubes budded from the pancake-like structure (see inset in Fig. 5a), and immunoreactive Rab11 was observed along the tube (Fig. 5b, arrow). To examine the relationship between the pancake-like structure and the elongated tube, we performed the live imaging analysis. Two pancake-like droplets (arrows) fused with each other within 18 min, and then a tube emerged from the fused droplet (arrow) and elongated (Fig. 5c and Supplementary Movie 1).

To identify the components of the pancake-like structures, we performed confocal Raman spectral measurement under fluorescence observation of GFP-JRAB/MICAL-L2. The pancake-like structures were recognized in the wide-field fluorescence microscopy image (Fig. 5d). Low-frequency Raman spectra averaged over the areas inside and outside the pancake-like structure revealed a distinct Raman band at 1654 cm^−1^ originating from C=O stretching vibration of amide groups, which is primarily attributed to proteins (Fig. 5e). The averaged intensity of the Raman band acquired inside the structure (i) was higher than the intensity outside the structure (ii) (1.35 ± 0.07, *n* = 3). Furthermore, in high-frequency Raman spectra, the intensity of the Raman band at 2935 cm^−1^ originating from the –CH_3_ stretching mode of proteins also differed between the interior and exterior of the structure in the same manner as the band at 1654 cm^−1^ (1.27 ± 0.07, *n* = 3), supporting the idea of protein condensates (Fig. 5f). These results suggest that the pancake-like structure is a droplet of the condensed proteins GFP-JRAB/MICAL-L2 and HA-Rab8ADA. Notably, 3D images revealed that the droplet was spherical and filled with GFP-JRAB/MICAL-L2 and HA-Rab8ADA (Fig. 5g). Immunogold electron microscopy revealed that both GFP-JRAB/MICAL-L2 and HA-Rab8ADA were present in confined areas in the cytoplasm of the transfected cells. These areas were more or less circular or oval in shape and comparable in size to the “pancakes” observed by immunofluorescence and live imaging, described above. They were not membrane-bound, and their interiors were unevenly electron-dense (Supplementary Fig. 7). These results led us to postulate that JRAB/MICAL-L2 and Rab8A induce liquid–liquid phase separation (LLPS).

Recent studies revealed that the condensed liquid droplets initiate various cellular functions, for example, regulation of transcription, autophagy, and the proteasome system^19,20^. Our findings raise the possibility that the condensed liquid droplets of JRAB/MICAL-L2 and Rab8A have a role in endosome tubulation. To examine the mobility of the condensed proteins between the droplets and surrounding solution, we monitored the fluorescence intensities of GFP-JRAB/MICAL-L2 using the FRAP assay. After bleaching, GFP signal within the region of interest (ROI) of the droplet recovered with a half-time of 24 s (Fig. 5h and Supplementary Movie 2). This exchange rate is compatible with that of molecules in various intracellular condensates^31^ and indicates that these droplets are not immobile aggregates consisting of the two proteins. We also examined pancake droplets induced by endogenous JRAB/MICAL-L2 using an anti-JRAB/MICAL-L2 antibody. The droplets were not observed in HeLa cells without exogenous JRAB/MICAL-L2 under the present experimental conditions (Supplementary Fig. 8), reasoning that a trigger may be needed to induce LLPS. In this study, overexpression of JRAB/MICAL-L2 and Rab8ADA may be a trigger for LLPS. Next, we verified that JRAB/MICAL-L2 and Rab8A could undergo LLPS in vitro using the purified recombinant proteins His-GFP-JRAB/MICAL-L2 and His-mCherry-Rab8ADA. Phase-separated droplets containing His-GFP-JRAB/MICAL-L2 and His-mCherry-Rab8ADA were observed in solution with the polymeric crowding agent PEG 5% (w/v) (Fig. 5i). Excluded volume effects mimic the intracellular crowding in the cytosol and allow proteins to achieve their critical concentrations. No droplets were observed in solutions of His-GFP-JRAB/MICAL-L2 and His-mCherry-Rab8ADA without crowding agent (Fig. 5i). In addition, we showed that the maximal diameter of some droplets could reach approximately 2.5 μm at 5 μM, and that the size of the droplets started decreasing at 2.5 μM. The saturation concentration above which His-GFP-JRAB/MICAL-L2 and His-mCherry-Rab8ADA were able to phase separate to spherical liquid droplets was less than 0.5 μM (Supplementary Fig. 9). These data indicated that JRAB/MICAL-L2 and Rab8ADA induce LLPS in vitro.

Taken together, these findings suggest that JRAB/MICAL-L2 and Rab8A drive the tubulation of recycling endosomes by inducing LLPS.

### The intrinsically disordered region of JRAB/MICAL-L2 is required for the initiation of the liquid–liquid phase separation

**Fig. 6:**
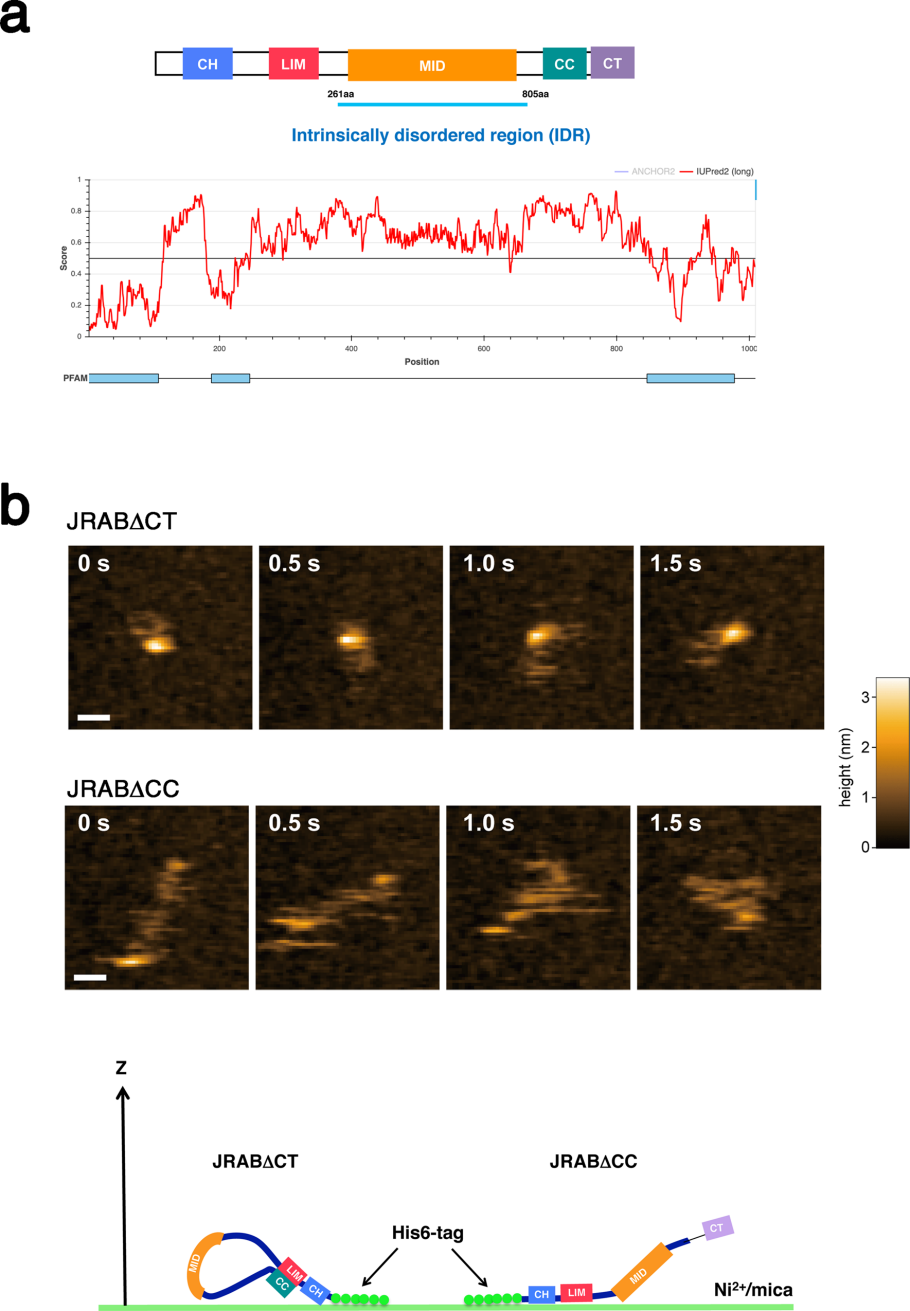
JRAB/MICAL-L2 contains a large IDR between N-terminal and C-terminal globular domains. **a** The schematic indicates the domain structure of JRAB/MICAL-L2 (upper). JRAB/MICAL-L2 was predicted by IUPred2A to contain a large IDR (score > 0.6) between the N-terminal and C-terminal globular domains (lower). **b** Typical HS-AFM images clipped from successive images of JRABΔCT and JRABΔCC immobilized on Ni^2+^/mica substrate at the N terminus. HS-AFM images were recorded at a frame rate of 10 fps, and images are shown every 0.5 s (see also Supplementary Movies 3 and 4). Scale bar, 20 nm. The schematic depicts putative conformations of JRABΔCT and JRABΔCC anchored on the Ni^2+^/mica substrate through a His6-tag at the N terminus. Histograms of the radius of gyration (*R*_g_) for JRABΔCT and JRABΔCC are shown in Supplementary Fig. 10. Experiments were repeated independently five times, with similar results.

We further examined the involvement of JRAB/MICAL-L2 protein in LLPS. PSPredictor (http://www.pkumdl.cn:8000/PSPredictor/) predicted that JRAB/MICAL-L2, but not Rab8A, can undergo LLPS. The electrostatic interaction between IDRs of intrinsically disordered proteins could explain their liquid-like condensates^19–21^. Indeed, according to the IUPred2A (https://iupred2a.elte.hu/), JRAB/MICAL-L2 is predicted to contain a large IDR between its N-terminal and C-terminal globular domains (Fig. 6a). To further characterize the IDR of JRAB/MICAL-L2, we observed recombinant proteins in the closed or open form using high-speed atomic force microscopy (HS-AFM), which revealed the behavior of JRAB/MICAL-L2 mutants in solution with high temporal resolution. HS-AFM images of JRABΔCT (closed form) immobilized by the His6-tag on a mica substrate covered by Ni^2+^ revealed that the two globules at the N-terminal and C-terminal were merged, and the string-like structure was constantly disordered and highly flexible (Fig. 6b and Supplementary Movie 3). On the other hand, in the images of JRABΔCC (open form), we observed the linkage of two separate globules by a highly flexible string-like structure (Fig. 6b and Supplementary Movie 4). To quantitatively compare the size compaction of JRABΔCT and ΔCC, we analyzed the radius of gyration (*R*_g_) of each molecule (Supplementary Fig. 10). Both the center value (~22 nm) and width (~7.4 nm) of the distribution of *R*_g_ for JRABΔCC were greater than those (the center value, ~11 nm; the width, ~3.3 nm) for JRABΔCT, indicating that JRABΔCC is in the open form, whereas JRABΔCT is in the closed form. Therefore, these findings clearly demonstrate that the middle domain of JRAB/MICAL-L2, between the N-terminal and C-terminal regions, is intrinsically disordered.

**Fig. 7:**
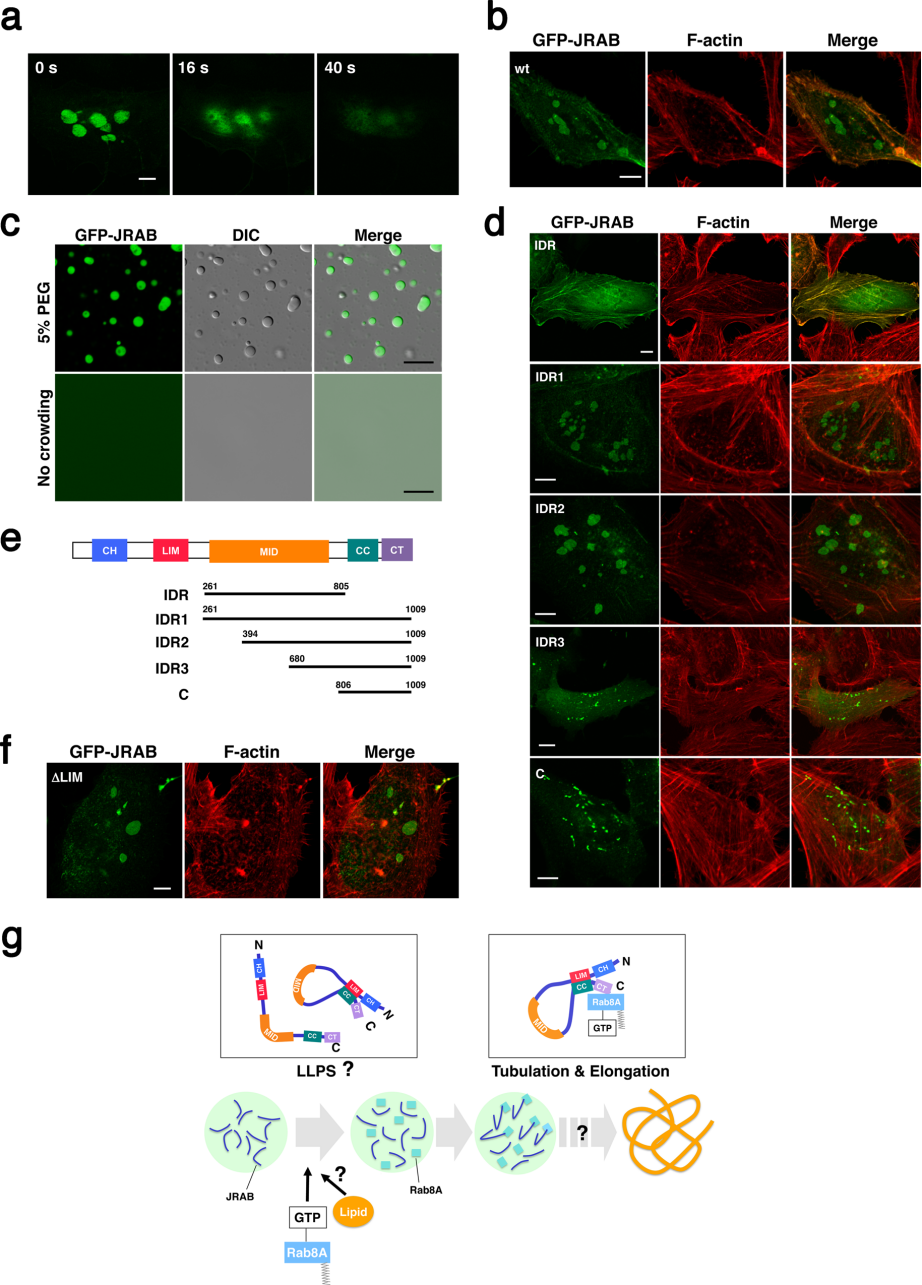
The intrinsically disordered region of JRAB/MICAL-L2 is required for the initiation of liquid–liquid phase separation. **a** Immediately after the addition of 1,6-hexanediol (10% w/v) to HeLa cells, the droplets of condensed GFP-JRAB/MICAL-L2 and HA-Rab8ADA disappeared. Images were captured at the indicated times (see also Supplementary Movie 5). Scale bar,10 μm. **b** Twenty-four hours after transfection, HeLa cells expressing GFP-JRAB (wt) were fixed, followed by staining with rhodamine–phalloidin. Note that GFP-JRAB alone could induce the droplets. Scale bar, 10 μm. **c** His-GFP-JRAB (green) exhibited liquid-like droplet formation in the absence of Rab8ADA by addition of crowding agent (upper panels, 5% PEG; lower panels, no crowding agent). Scale bar, 10 μm. **d** Twenty-four hours after transfection, HeLa cells expressing GFP-JRAB IDR (aa 261–805), GFP-JRAB IDR1 (IDR plus C terminus [aa 261–1009]), IDR2 (large part of IDR plus C terminus [aa 394–1009]), IDR3 (small part of IDR plus C terminus [aa 680–1009]), or C (aa 806–1009) were fixed and stained with rhodamine–phalloidin. The schemas of each mutant are shown in **e**. Scale bar, 10 μm. **e** The schemas of each JRAB mutant. **f** Twenty-four hours after transfection, HeLa cells expressing GFP-JRABΔLIM were fixed and stained with rhodamine–phalloidin. Scale bar, 10 μm. **g** Model of LLPS and formation of tubular recycling endosomes via the JRAB/MICAL-L2–Rab8A complex. JRAB/MICAL-L2 (open form or closed form) undergoes LLPS in the absence of Rab8A. Rab8A may supply lipid components derived from endosomes to the droplet of condensed JRAB/MICAL-L2. Tubulation of recycling endosomes is initiated from the liquid-like droplet containing closed-form JRAB/MICAL-L2 associated with Rab8A. Thus, the conformational change of JRAB/MICAL-L2 via Rab8A is important for the transition from LLPS to tubulation of recycling endosomes. Experiments were repeated independently three times, with similar results (**a**, **c**). More than 40 transfected cells from four individual preparations were examined, and representative images are shown (**b**, **d**, **f**).

Thus, it is likely that the IDR of JRAB/MICAL-L2 contributes to the formation of pancake-like structure. To test that idea, we examined the effect of 1,6-hexanediol on condensates of GFP-JRAB/MICAL-L2 and HA-Rab8ADA. 1,6-hexanediol is an inhibitor of interactions between IDRs^32^. The droplets disappeared in the presence of 1,6-hexanediol (Fig. 7a and Supplementary Movie 5). The results indicate that IDR of JRAB/MICAL-L2 is responsible for the LLPS.

Next, we examined the formation of a pancake-like structure in HeLa cells expressing GFP-JRAB/MICAL-L2 alone. GFP-JRAB/MICAL-L2 induced the structures even in the absence of Rab8ADA (Fig. 7b). Moreover, in vitro experiments revealed that His-GFP-JRAB/MICAL-L2 alone formed a liquid-like droplet upon addition of PEG (Fig. 7c). These results suggest that JRAB/MICAL-L2 induces LLPS without Rab8ADA in cellulo and in vitro. To determine the exact region responsible for the induction of LLPS, we further prepared GFP-JRAB IDR (aa 261–805), GFP-JRAB IDR1, containing the whole IDR plus the C terminus (aa 261–1009), GFP-JRAB IDR2, containing a large part of the IDR plus the C terminus (aa 394–1009), and GFP-JRAB IDR3, containing a small part of the IDR plus the C terminus (aa 680–1009), in addition to GFP-JRAB-C (aa 806–1009), and examined their effect on the formation of liquid-like droplets (Fig. 7e). The pancake-like structures were observed in cells expressing GFP-JRAB IDR1 or GFP-JRAB IDR2, but not in those expressing GFP-JRAB IDR, GFP-JRAB IDR3, or GFP-JRAB-C (Fig. 7d). These cell biological data indicated that not only the IDR of JRAB/MICAL-L2, but also the C terminus, is involved in the formation of liquid-like droplets. Here, it should be noted that GFP-JRAB IDR1 and GFP-JRAB IDR2 lack the intramolecular interaction between the N terminus and C terminus; i.e., the mutants are in the open form. On the other hand, full-length GFP-JRAB mainly adopts the closed form, as we showed previously^17^. From these findings, we concluded that JRAB/MICAL-L2 induces LLPS via the IDR and C terminus regardless of its conformation. Indeed, GFP-JRABΔLIM, which adopts the open form and contains both the IDR and C terminus, also formed liquid-like droplets (Fig. 7f).

Based on these observations, we propose the following mechanism of the tubulation of recycling endosomes (Fig. 7g). First, condensed JRAB/MICAL-L2 forms liquid-like droplets. Then, Rab8A is recruited to the droplets and provides lipids to enable the formation of tubular recycling endosomes. In the droplet, Rab8A keeps JRAB/MICAL-L2 in the closed form, as shown in Fig. 1. Closed-form JRAB/MICAL-L2–Rab8A complex could be the trigger for initiation of tubulation of recycling endosomes, subsequently contribute to the formation of long and complicated tubular structures.

## Discussion

In the present study, we propose that two kinds of Rab proteins fulfill their individual functions through a conformational change of their common effector in the corresponding sites within the cell. The results of this study provide the first evidence that JRAB/MICAL-L2, along with Rab8A, is involved in the membrane dynamics of organelles. The target organelle is the recycling endosome, which functions in the recycling of various molecules (e.g., adhesion molecules and receptors) to the plasma membrane. Recycling endosomes change their morphology from punctate to tubular structures, and these dynamics are necessary for cargos to reach their individual destinations. Here, we provided the data suggesting that tubulation of recycling endosomes is induced by JRAB/MICAL-L2–Rab8A complex. We also found that Rab8A allows JRAB/MICAL-L2 to adopt the closed form. Moreover, experiments using two chimeric variants of Rab13 and Rab8A, in which the C-terminal variable regions were reciprocally swapped, revealed that the conformation of JRAB/MICAL-L2 is determined by the C terminus of Rabs, including the cysteine residue modified by lipids. Based on our findings, we conclude that the conformation of JRAB/MICAL-L2 changes closed form through association with Rab8A, and that the interaction of the closed form of JRAB/MICAL-L2 and Rab8A induces membrane dynamics, including tubulation of recycling endosomes.

JRAB/MICAL-L2 belongs to the MICAL family^16^. MICAL-L1, another MICAL family protein, has a pivotal role in the tubulation of recycling endosome^18^. MICAL-L1 interacts with many regulatory proteins including syndapin2, EHD-1, and Rab8A and recruits them to tubular recycling endosomes^6^. However, MICAL-L1 forms a tubular endosome structure in the absence of Rab8A. Recently, Fukuda’s group reported that Rab10 forms MICAL-L1-positive tubular endosomes in a microtubule-dependent manner^5^. In that study, they showed that the tubules induced by Rab10 were still observed in Rab8A/B double-knockout cells. Consequently, the role of Rab8A in the tubulation of recycling endosome had remained unknown. Here, we presented the data suggesting that Rab8A contributes to the formation of a long and complicated tubular structure of recycling endosomes in collaboration with JRAB/MICAL-L2. Moreover, we proposed that LLPS is responsible for endosomal tubulation via JRAB/MICAL-L2–Rab8A complex. Recently, LLPS has attracted a great deal of research interest^20,21,33–35^. Liquid-like droplets of proteins are involved in multiple cellular functions, including regulation of transcription, autophagy, and proteasome-mediated protein degradation^36–38^. Research on the significance of LLPS is essential for understanding various biological processes. Our present data suggested that JRAB/MICAL-L2 undergoes LLPS via its IDR and C terminus, followed by the tubulation of recycling endosomes. Phase-separated droplets of JRAB/MICAL-L2 were observed in the absence of Rab8A (Fig. 7b, c). Moreover, JRAB/MICAL-L2 formed the liquid-like condensate regardless of its conformation. On the other hand, in cells expressing JRAB/MICAL-L2 and Rab8ADA, Rab8ADA was observed in JRAB/MICAL-L2–positive liquid-like droplets. What, then, is the role of Rab8A in the LLPS induced by JRAB/MICAL-L2? We postulate that Rab8A supplies the lipid components derived from endosomes into droplets of condensed JRAB/MICAL-L2 (Fig. 7g). The possibility is consistent with the result of Raman spectral measurement, which revealed that the -CH_2_ stretching mode at 2851 cm^−1^, predominantly attributed to lipids, is more intense inside than outside the droplet (1.84 ± 0.14, *n* = 3), indicating that lipid molecules were being recruited into the liquid-like droplet (Fig. 5f). Furthermore, in the case without HA-Rab8ADA, the intensity of the lipid-attributed Raman mode was enhanced only by 1.19 ± 0.03 (*n* = 4) in the droplets of GFP-JRAB/MICAL-L2, revealing the crucial role of HA-Rab8ADA in recruiting lipid molecules (Supplementary Fig. 11). However, many questions remain. First, what allows JRAB/MICAL-L2 to undergo LLPS? Second, what is the trigger for the tubulation of recycling endosomes from liquid-like condensate of JRAB/MICAL-L2 and Rab8A? In this study, the expression level of JRAB/MICAL-L2 and Rab8ADA might reach the critical concentration by their overexpression, leading to LLPS in cellulo. In the in vitro assay, JRAB/MICAL-L2 or JRAB/MICAL-L2-Rab8ADA forms droplets by addition of PEG, which mimics a crowded intracellular environment. Future studies should seek to identify physiological triggers for LLPS or endosomal tubulation. In preliminary experiments, we detected tubulation of recycling endosomes induced by the JRAB/MICAL-L2-Rab8A complex during a Ca^2+^ switch assay in epithelial cells expressing moderate levels of GFP-JRAB/MICAL-L2 and mCherry-Rab8ADA via the retrovirus expression system. This finding supported the physiological significance of the formation and function of tubular endosomes induced by the JRAB/MICAL-L2-Rab8A complex. Third, how does the JRAB/MICAL-L2–Rab8A complex form a long and complicated tubular structure? Understanding the arranging and orientation of the JRAB/MICAL-L2–Rab8A complex on the tubular endosome could provide insight into this issue. Last, what is the factor that inhibits the tubulation of recycling endosomes regulated by the JRAB/MICAL-L2–Rab8A complex? Under physiological conditions, vesiculation should occur following tubulation of recycling endosomes. In the case of MICAL-L1, EHD-1, a dynamin-like molecule, has a primary role in the vesiculation of recycling endosomes, but we have not yet found any counterpart to EHD-1 for JRAB/MICAL-L2. In this study, we could not examine the process after tubulation because we used a dominant-active form of Rab8A. Therefore, we speculate as follows. When the tip of tubular endosome approaches its destination at the plasma membrane, JRAB/MICAL-L2 is transferred from Rab8A to Rab13, which localizes on the plasma membrane. On the plasma membrane, JRAB/MICAL-L2 can adopt its open form via interaction with Rab13, resulting in the inhibition of elongation of tubes. Consequently, vesiculation is induced, and cargo will be delivered to the correct destination. To address the questions enumerated above, further investigation will be required. The interdisciplinary approach using novel techniques and methods will help reveal the answers in future studies.

## Methods

### Plasmid construction

Mouse JRAB/MICAL-L2 mutants JRAB IDR1 (aa 261–1009), JRAB IDR2 (aa 394–1009), and JRAB IDR3 (aa 680–1009) were amplified by PCR using pCIneo-HA-JRAB/MICAL-L2 as a template. To generate EGFP constructs, JRAB-IDR (aa 261–805), JRAB-C (aa 806–1009), JRAB IDR1, JRAB IDR2, and JRAB IDR3 were subcloned into pEGFP-C1. pEGFP-JRABwt, -JRABΔCT, -JRABΔLIM, and pCIneoMyc-JRABwt were described previously^26,39^. For production of hexahistidine (His6)-tagged recombinant proteins, cDNAs encoding JRABΔCC and JRABΔCT were subcloned into pcDNAHisMax^17^. pGEX-6P-1-JRAB-CH+LIM and -C were described previously^17,40^, and pGEX-6P-1-JRAB-CC4 (aa 840–930) was generated in the same way. cDNAs encoding the dominant-active and dominant-negative mutants of mouse Rab13 (Rab13DA, Q67L) and mouse Rab8A (Rab8ADA, Q67L and Rab8ADN, T22N) were subcloned into pCIneo-HA, pEGFP-C1, or pmCherry-C1. Mouse Rab8ADA truncation mutants Rab8DAΔC1 (aa 1–166) and Rab8DAΔC3 (aa 1–190) were amplified by PCR using pEGFP-Rab8ADA as a template. The products were subcloned into pCIneo-HA or pEGFP-C1. pCIneoHA-Rab8ADA-C13 and pCIneoHA-Rab13DA-C8A were constructed using In-Fusion® HD Cloning Kit (Takara Bio). To generate EGFP constructs, cDNAs of the chimeric variants were amplified by PCR using pCIneo-HA constructs as a template. The products were subcloned into pEGFP-C1. To generate expression plasmid encoding the His6-tag followed by GFP or mCherry fluorescence protein, GFP or mCherry was amplified by PCR and cloned into pcDNAHisMax. cDNAs encoding JRAB/MICAL-L2 and Rab8ADA were subcloned into pcDNAHisMax-GFP and pcDNAHisMax-mCherry, respectively. All plasmids constructed in this study were sequenced using an ABI Prism 3100 genetic analyzer (Applied Biosystems).

### Cell culture

HeLa cells were cultured in Dulbecco’s modified Eagle’s medium (DMEM) with 10% fetal bovine serum (FBS), 100 U/ml penicillin, and 100 μg/ml streptomycin. HEK293 cells were cultured in DMEM with 5% FBS, 1 mM sodium pyruvate, 100 U/ml penicillin, and 100 μg/ml streptomycin. Cells were maintained at 37 °C in a water-saturated atmosphere of 95% air and 5% CO_2_.

### Pulldown and immunoprecipitation assays

HEK293 cells were seeded at a density of 5 × 10^5^ cells on 60-mm dishes and transfected the following day with the appropriate amount of each plasmid using the PEI-Max transfection reagent. After incubation for 48 h at 37 °C, the cells were washed once, scraped from the dishes in PBS, and lysed in 300 μl buffer A (10 mM Tris-HCl at pH 8.0, 1 mM EDTA, 1%[w/v] Nonidet P-40, 150 mM NaCl, 5 mM MgCl_2_, and 10 μM p-APMSF). The cell lysates were centrifuged at 4 °C for 5 min at 16,100×*g*, and each supernatant was incubated with various purified GST-JRAB/MICAL-L2 mutants attached to glutathione–Sepharose beads (for pulldown assays) or anti-HA antibody (12CA5) attached to Protein G beads (for immunoprecipitation assays) and incubated for 90 min at 4 °C. The beads were then washed with buffer A three times and resuspended in sodium dodecyl sulfate (SDS) sample buffer. Comparable amounts of proteins that remained associated with the beads were separated by SDS-PAGE. The fraction of target protein bound to the affinity column was determined by western blotting using anti-GFP, rat anti-HA, or mouse anti-Myc antibodies.

### Immunocytochemistry and confocal laser scanning microscopy

HeLa cells were seeded at a density of 2 × 10^5^ cells on coverslips in individual 35-mm dishes or in 35-mm glass-bottom dishes. The following day, the cells were transfected with the indicated plasmid(s) and cultured for 1 or 2 days. Then, the cells were fixed with 1 or 4% paraformaldehyde (PFA) in PBS at room temperature for 20 min. After washing with PBS, the cells were incubated in 5% donkey serum and 0.1% Triton X-100 in PBS for 30 min. Next, cells were incubated with primary antibodies for 1 h, followed by incubation with fluorescent dye-conjugated secondary antibodies (Jackson, Thermo Fisher) for 1 h. F-actin was labeled with rhodamine–phalloidin (Thermo Fisher). Coverslips were mounted on glass slides, and samples were observed using an A1 confocal laser scanning microscope (Nikon) equipped with a dry objective lens (PlanApo ×40, NA = 0.95 or PlanApo VC ×60, NA = 1.4) and excitation lasers (488, 561, and 640 nm, COHERENT).

### FRAP assay

Fluorescence recovery after photobleaching (FRAP) was performed as described elsewhere^36,41^. In brief, circular ROIs (7.56 μm^2^) were intensively illuminated with 488 nm laser for 5 s (1 repeat, 100% transmission: ~25 mW laser power) to reduce the fluorescence signals. Images acquisition was performed every 1 s for 5 min. Data were obtained from three independent experiments. A single exponential fit to the FRAP data in ORIGN8 (Origin Lab) on time-averaged data (5 frames) was used to estimate the recovery half-time.

### Immunoelectron microscopy

Transfected cells were fixed for 5 min at room temperature (RT) with 1% formaldehyde + 0.1% glutaraldehyde (GA) in 0.1 M phosphate buffer (pH 7.4), harvested from the culture dish, and recovered by centrifugation in an Eppendorf tube. Cell pellets were fixed for an additional 4 h in the same fixative and changed to 8% sucrose + 0.5% glycine in 0.1 M phosphate buffer (pH 7.4). The samples were dehydrated in an ascending ethanol series and embedded in LR White (London Resin Company). Ultrathin sections were blocked for 1 h at RT with 1% BSA in 0.1 M phosphate buffer, and then stained at 4 °C overnight with rabbit polyclonal anti-GFP(FL) antibodies (1:75, cat# sc-8334; Santa Cruz Biotechnology) or rabbit polyclonal anti-HA antibodies (1:30, cat# sc-805; Santa Cruz Biotechnology). The sections were then incubated at RT for 1.5 h with goat anti-rabbit IgG conjugated colloidal gold (1:50, 10 nm: cat# EMGAR10, 20 nm: cat# EMGAR20; BBI Solutions, Cardiff, UK) or protein A-conjugated 10 nm colloidal gold, prepared as described (Slot and Geuze, 1985). In some cases, sections were counterstained with uranyl acetate and lead citrate and observed on a Hitachi H-7650 transmission electron microscope (Hitachi High-Tech).

### Western blotting

Cell lysates, immunoprecipitates, or pulled-down proteins were separated by SDS-PAGE, transferred to Immobilon-P membranes, and blocked for 1 h in Tris-buffered saline containing 5% nonfat dry milk. After incubation with specific primary antibodies for 1 h, followed by HRP-coupled secondary antibodies (Jackson) for 1 h, immunoreactive proteins were visualized using Western Blotting Substrate Plus (Pierce).

### Bacterial production of recombinant proteins

GST-fusion proteins were expressed in *Escherichia coli* (*E. coli*) strain DH5α cultured in the presence of 0.1 mM isopropyl-β-d-thiogalactopyranoside. Bacteria were resuspended in buffer B (20 mM Tris-HCl at pH 8.0, 1 mM EDTA, 1 mM dithiothreitol, and 10 μM p-APMSF) and lysed by sonication, and cellular debris was removed by centrifugation for 60 min at 100,000×*g*. The resultant supernatant was applied to the glutathione–Sepharose 4B column (GE Healthcare Biosciences). The pass-through fraction was applied a second time to the same column. The column was washed with buffer B, and GST-fusion proteins were eluted in buffer C (50 mM Tris-HCl at pH 8.0 and 10 mM reduced glutathione). The eluted proteins were detected by electrophoresis and Coomassie Brilliant Blue (CBB) staining.

### Purification of recombinant proteins from HEK293 cells

HEK293 cells were seeded at a density of 1.5 × 10^6^ cells on 100-mm dishes and transfected the following day with 12 μg of each plasmid using PEI-Max transfection reagent. After a 48-h incubation at 37 °C, the cells were washed once and scraped from the dishes in PBS. The cells were lysed in 300 μl buffer D (10 mM Tris-HCl at pH 8.0, 1 mM EDTA, 1% [w/v] Nonidet P-40, 150 mM NaCl, and 10 μM p-APMSF) for His-JRABΔCC, His-JRABΔCT, and His-GFP-JRAB and then the cell lysates were centrifuged at 4 °C for 5 min at 16,100×*g*. The supernatants collected from 30 dishes were applied to a cOmplete resin column (Roche). The pass-through fraction was applied to the same column a second time. The column was washed with buffer E (50 mM Tris-Cl at pH 7.5, 150 mM NaCl, 2 mM DTT, and 10 mM imidazole). The proteins remaining in the column were eluted with buffer F (50 mM Tris-Cl at pH 7.5, 150 mM NaCl, 2 mM DTT, and 100 mM imidazole) and subjected to SDS-PAGE. The samples were concentrated, and then 500 μl of the protein solution was applied to a Superdex 200 PC 3.2/30 (GE Healthcare) column (2.6 cm × 6.6 cm) equilibrated with buffer G (25 mM Tris-HCl at pH 7.5, 0.5 mM EDTA, 1 mM DTT, and 150 mM NaCl). Elution was performed with buffer G at a flow rate of 250 μl/min. Fractions were collected and subjected to SDS-PAGE, followed by CBB staining. For His-mCherry-Rab8ADA, cells collected from 60 dishes were sonicated in 25 ml buffer H (20 mM Tris-HCl at pH 8.0, 5 mM MgCl_2_, 100 mM NaCl, and 10 μM p-APMSF), and then the homogenates were centrifuged at 4 °C for 1 h at 100,000×*g*. The pellet fractions were extracted with buffer I (20 mM Tris-HCl at pH 8.0, 5 mM MgCl_2_, 100 mM NaCl, 1% CHAPS, and 10 μM p-APMSF) and centrifuged at 4 °C for 1 h at 100,000×*g*. The supernatants (extracted fractions) were applied to a Talon resin column (Clontech). The pass-through fraction was applied to the same column a second time. The column was washed with buffer J (20 mM Tris-Cl at pH 8.0, 5 mM MgCl_2_, 100 mM NaCl, 0.6% CHAPS, and 10 mM imidazole). The proteins remaining in the column were eluted with buffer K (20 mM Tris-Cl at pH 8.0, 5 mM MgCl_2_, 100 mM NaCl, 0.6% CHAPS, and 100 mM imidazole) and subjected to SDS-PAGE.

### Evaluation of JRAB indicator in vitro

The FRET-based JRAB indicator was constructed by assembling cDNA fragments encoding Venus, full-length JRAB/MICAL-L2, and cp173ECFP into pRSET-B and subcloned into pcDNA4-HMB, as previously described^17,42^. HEK293 cells transiently expressing JRAB indicator along with HA-tagged construct were collected and lysed in buffer A. Cell lysates were then directly subjected to spectral measurement on an FP-6300 spectrofluorometer (Jasco). The FRET emission was defined as the ratio of the YFP/CFP (excitation at 433 nm, emission at 476 and 527 nm). The high- and low-ratio values presumably correspond to the statuses of JRAB/MICAL-L2 in the closed and open forms, respectively.

### Raman spectral measurement

Raman spectral measurement of PFA-fixed HeLa cells in PBS was performed using a confocal Raman microscope (Raman 11, Nanophoton) with an excitation wavelength of 532 nm. The spatial resolution of the microscope was ~330 nm with a water-immersion objective lens (×60, NA = 1.0). The laser power density was set to 0.3 W/cm^2^ during Raman spectral mapping to avoid laser-induced damage to the biological sample. The Raman microscope was combined with a wide-field fluorescence microscope, enabling us to spatially identify the GFP-JRAB/MICAL-L2-positive structures in Raman measurements.

### High-speed atomic force microscopy (HS-AFM)

To visualize JRAB/MICAL-L2 molecules, a laboratory-built HS-AFM with tapping mode was used to minimize possible perturbation to the sample by imaging^43^. We used a miniaturized cantilever developed by Olympus (AC7), with nominal spring constant, resonant frequency, and quality factors of ~0.2 N/m, ~0.8 MHz, and ~2, respectively, in solution. Free oscillation of the cantilever for the tapping motion was ~1 nm, and the amplitude was reduced to ~90% of free amplitude on the sample for feedback control. To gain a sharp AFM probe, a carbon pillar was deposited on the blunt beak-like structure on the cantilever end by electron beam deposition^44^. After deposition, the carbon tip was sharpened by Ar-ion sputtering. These etching processes gave a sharp tip with an end radius less than 4 nm. As a substrate, we used a freshly cleaved mica with Ni^2+^ coverage. Just after the mica was cleaved, a droplet of 2 mM NiSO_4_ solution was placed on the bare mica substrate. Ni^2+^ bound to the mica substrate allowed an N-terminally His6-tagged JRAB protein to be anchored onto the substrate due to chelation of Ni^2+^ by the tag^45^. After incubation for 3 min, the substrate was washed with pure water. A sample solution containing JRABΔCC (final concentration: 45 nM) or JRABΔCT (final concentration: 40 nM) was deposited on the Ni^2+^/mica substrate. After incubation for 3 min, residual proteins that were not tightly adsorbed on the substrate were washed away with buffer G. HS-AFM imaging was performed at room temperature under the buffer G.

To assess the spatial compaction of JRAB molecules based on HS-AFM images, the radius of gyration of the molecule was measured. First, the area of image contrast corresponding to a single molecule was determined by an appropriate threshold. Second, the center of gravity position $({x}_{\mathrm{g}},{y}_{\mathrm{g}})$ was found based on the height at each pixel within that area. Then the radius of gyration (*R*_g_) was calculated by the following formula:

$${R}_{\mathrm{g}}=\sqrt{\frac{\mathop{\sum}\limits_{i,j}{I}_{{ij}}\left\{{\left({x}_{{ij}}-{x}_{\mathrm{g}}\right)}^{2}+{\left({y}_{{ij}}-{y}_{\mathrm{g}}\right)}^{2}\right\}}{\mathop{\sum}\limits_{i,j}{I}_{{ij}}}}$$

Here, ${I}_{{ij}}$ and $({x}_{{ij}},{y}_{{ij}})$ are height and position at pixel point $(i,{j})$ in the area corresponding to a single molecule, respectively. *R*_g_ is considered to be an indicator of molecular compaction.

### Liposomes experiments

Liposomes were prepared using L-α-phosphatidylcholine (brain PC, porcine) and L-α-phosphatidylserine (brain PS, porcine) purchased from Avanti Polar Lipids (Alabaster, AL, USA); these lipids were used without further purification. Each lipid was solubilized in chloroform at a concentration of 10 mM to make a stock solution. Then, the stock solutions of brain PC and brain PS were mixed and diluted in chloroform to 0.7 and 0.3 mM, respectively. The solvent of the lipid mixture was evaporated under a flow of nitrogen gas for 1 h. The dried lipids were suspended in 0.3 M sucrose at 37 °C for 1 h, followed by vortexing (final concentration: 1 mM). The liposome suspensions were diluted 20-fold with hypotonic buffer L (20 mM HEPES-NaOH, pH 7.5, 100 mM KCl, 1 mM DTT) to obtain large liposomes (typically 1–10 μm in diameter), and then mixed with purified His-GFP-JRAB/MICAL-L2 and His-mCherry-Rab8ADA (final concentration: 150 nM). The mixture was placed on a glass-bottom dish, covered to avoid evaporation, and observed using an A1 confocal laser scanning microscope (Nikon) equipped with an oil objective lens (PlanApo VC ×60, NA = 1.4).

### Formation of phase-separated droplets with crowding agents

To observe the droplets, 25% of PEG (final concentration: 5%) was added to a mixture of purified His-GFP-JRAB/MICAL-L2 with or without His-mCherry-Rab8ADA. The mixture was placed on a glass-bottom dish. Images were acquired using an A1 confocal laser scanning microscope (Nikon) equipped with an oil objective lens (PlanApo VC × 60, NA = 1.4).

### Statistics and reproducibility

One-way analysis of variance (ANOVA) followed by Tukey’s post-hoc test was used for multiple group comparisons. When necessary, Welch’s *t*-test followed by Dunnett’s post-hoc test was used for unequal variances. Differences were assessed with a two-sided test and considered significant when *p* < 0.05.

### Reporting summary

Further information on research design is available in the Nature Research Reporting Summary linked to this article.

## Supplementary information

Supplementary Information

Description of Additional Supplementary Files

Supplementary Movie 1

Supplementary Movie 2

Supplementary Movie 3

Supplementary Movie 4

Supplementary Movie 5

Supplementary Data 1

Reporting Summary

## Data Availability

All data generated or analyzed during this study are included in this published article (and its supplementary information files). Resources used in this study are provided in Supplementary Table 1. The source data for the main figures is provided in Supplementary Data 1. Any remaining information can be obtained from the corresponding author upon reasonable request.
