# Supplementary material for: JRAB/MICAL-L2 undergoes liquid–liquid phase separation to form tubular recycling endosomes
Source: Commun Biol. 2021 May 11;4:551. doi: 10.1038/s42003-021-02080-7 (PMC8113518; doi:10.1038/s42003-021-02080-7)
Supplement: Supplementary file 1 — Supplementary Information [file 42003_2021_2080_MOESM1_ESM.pdf]

Supplementary Information for

JRAB/MICAL-L2 undergoes liquid–liquid phase separation to form tubular recycling endosomes

Ayuko Sakane, Taka-aki Yano, Takayuki Uchihashi, Kazuki Horikawa, Yusuke Hara, Issei Imoto, Shusaku Kurisu, Hiroshi Yamada, Kohji Takei, and Takuya Sasaki

Supplementary Fig.1

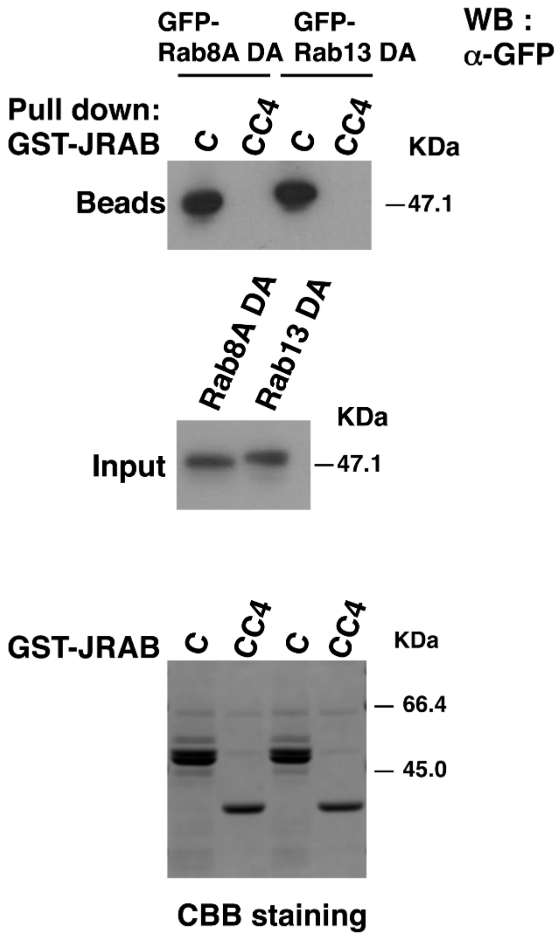

Supplementary Fig. 1. Rab8ADA and Rab13DA binds to JRAB-C, but not JRAB-CC4, related to Fig. 1b. Lysates of HEK293 cells expressing GFP-Rab8ADA or GFP-Rab13DA were subjected to pulldown assay using GST-JRAB-C (aa 806–1,009) or GST-JRAB-CC4 (aa 840–930). Pulled-down GFP-Rab8ADA or GFP-Rab13DA (Beads) was detected by western blotting (WB) with anti-GFP antibody. Total cell lysates (Input) were

also analyzed with anti-GFP antibody. The amount of GST-JRAB-C or GST-JRAB-CC4 attached to glutathione–Sepharose beads was determined by SDS-PAGE followed by CBB staining. Experiments were repeated independently three times, with similar results. Uncropped blots and gel are shown in Supplementary Fig. 12.

Supplementary Fig.2

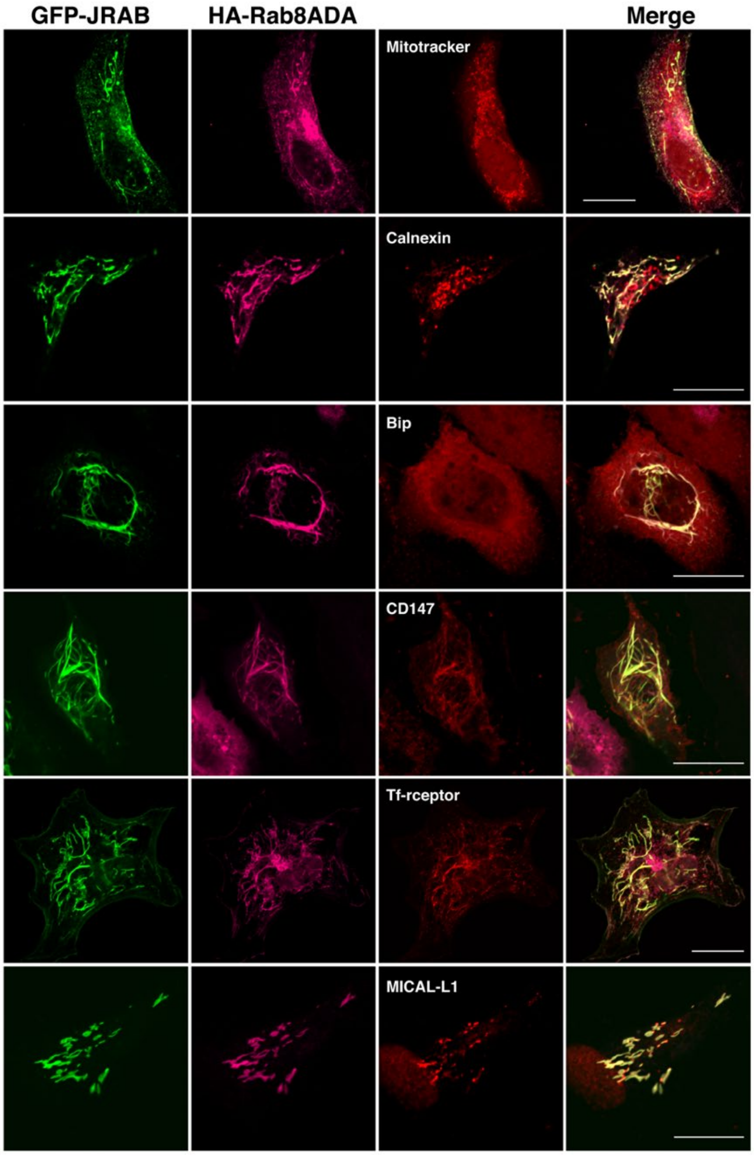

Supplementary Fig. 2. Localization of organelle markers on the tubules coated with GFP-JRAB/MICAL-L2 and HA-Rab8ADA, related to Fig. 2. HeLa cells transfected

with HA-Rab8ADA and GFP-JRAB/MICAL-L2 were fixed and double-stained with an anti-HA (magenta) antibody and antibody for the indicated marker (red). Scale bar, 20 μm. More than 40 transfected cells from four individual preparations were examined, and representative images are shown.

Supplementary Fig.3

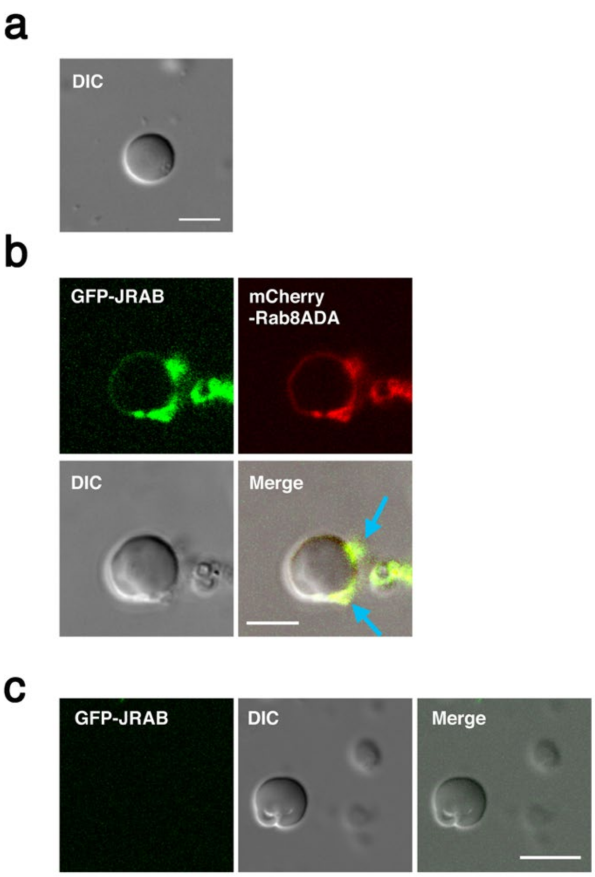

Supplementary Fig. 3. JRAB/MICAL-L2 deformed liposomal membrane along with Rab8A, related to Fig. 2. a. Liposomes consisting of a representative neutral phospholipid (PC) and an acidic phospholipid (PS) (7:3 mol/mol). Scale bar, 5 μm. **b.** His-GFP-JRAB/MICAL-L2 (green) and His-mCherry-Rab8ADA (red) were added to the liposomes. The final concentrations of His-GFP-JRAB/MICAL-L2 and His-mCherry-Rab8ADA were 0.15 μM. Scale bar, 5 μm. *Blue arrows* indicate bud-like structures of

liposomal membranes. **c.** His-GFP-JRAB/MICAL-L2 (green) was added to the liposomes. The final concentration of His-GFP-JRAB/MICAL-L2 was 0.15 μM. Scale bar, 10 μm. Experiments were repeated independently three times, with similar results.

Supplementary Fig.4

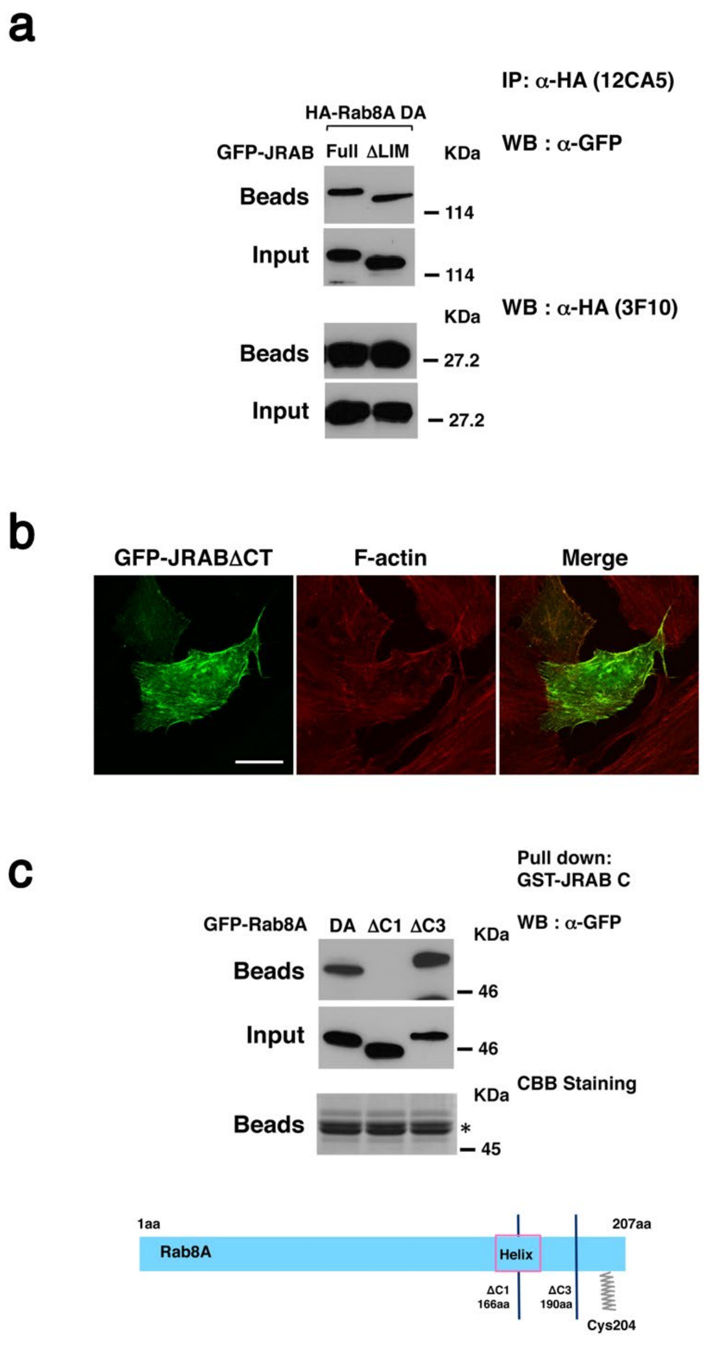

Supplementary Fig. 4. Association of closed-form JRAB/MICAL-L2 with Rab8A and C-terminus of Rab8A are necessary for endosomal tubulation, related to Fig. 3.

a. Lysates of HEK293 cells co-expressing HA-Rab8ADA with GFP-JRAB/MICAL-L2 (Full) or GFP-JRABΔLIM were subjected to immunoprecipitation with anti-HA antibody (12CA5). Immunoprecipitates (Beads) and total cell lysates (Input) were detected by western blotting (WB) with anti-GFP and anti-HA antibodies (3F10). **b.** HeLa cells transfected with GFP-JRABΔCT were fixed and stained with rhodamine–phalloidin. Scale bars, 25 μm. **c.** Lysates of HEK293 cells expressing GFP-Rab8ADA, Rab8ADAΔC1, or Rab8ADAΔC3 were subjected to pulldown assays using GST-JRAB-C. Pulled-down proteins (Beads) and expressed protein in total cell lysates (Input) were detected by western blotting (WB) with anti-GFP antibody. The amount of GST-JRAB-C attached to glutathione–Sepharose beads was determined by SDS-PAGE, followed by CBB staining (*asterisk*). The schematic summarizes the sequence structure of the Rab8A truncated mutants. Cys204 represents the position of amino acid with a geranylgeranyl modification. Experiments were repeated independently three times, with similar results (a, c). More than 40 transfected cells from four individual preparations were examined and representative images are shown (b). Uncropped blots and gel are shown in Supplementary Fig. 12 (a, c).

Supplementary Fig.5

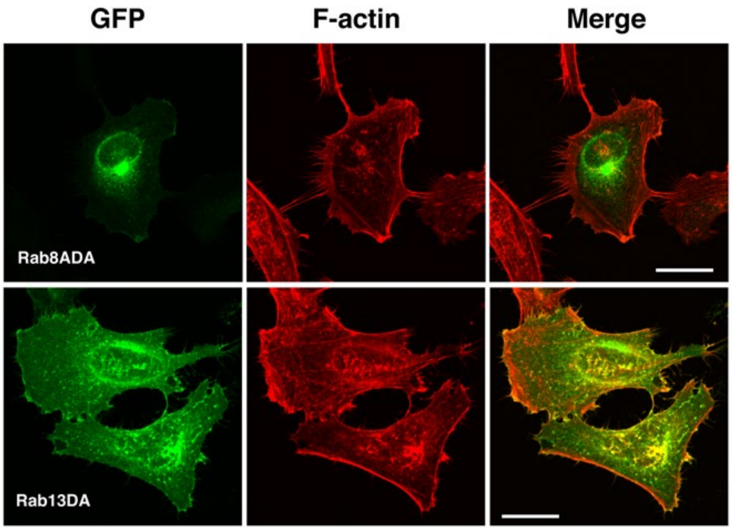

Supplementary Fig. 5. Localization of GFP-Rab8ADA and GFP-Rab13DA in HeLa Cells, related to Fig. 4b. HeLa cells expressing GFP-Rab8ADA or GFP-Rab13DA were fixed and stained with rhodamine–phalloidin. Scale bars, 25 μm. GFP-Rab8ADA localized at perinuclear regions, whereas GFP-Rab13DA localized at the plasma membrane. More than 40 transfected cells from four individual preparations were examined, and representative images are shown.

Supplementary Fig.6

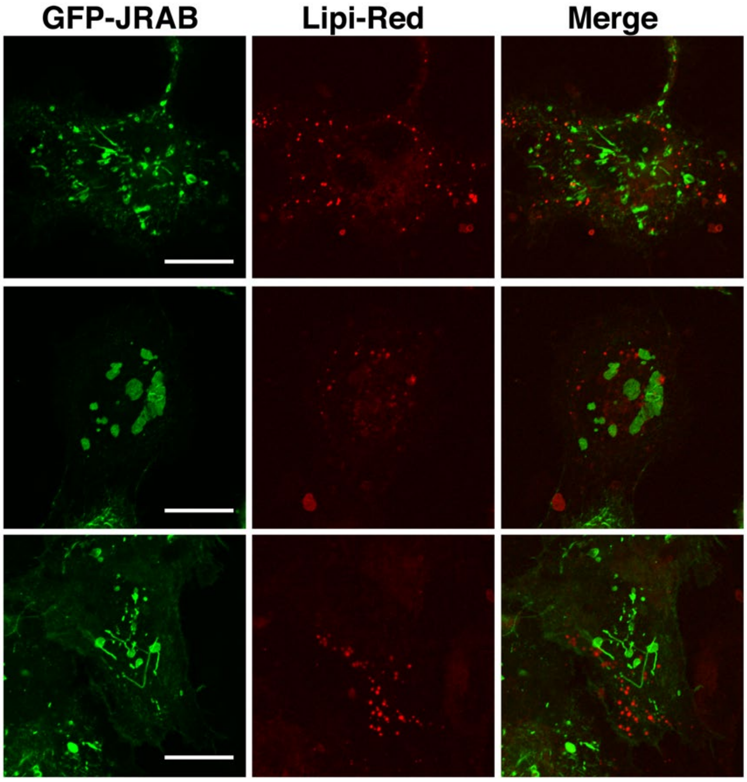

Supplementary Fig. 6. Marker for lipid droplets was not localized on the pancake-like structure, related to Fig. 5a. HeLa cells transfected with HA-Rab8ADA and GFP-JRAB/MICAL-L2 were fixed and stained with Lipi-Red (red). Scale bar, 20 µm. More than 40 transfected cells from four individual preparations were examined, and representative images are shown.

Supplementary Fig.7

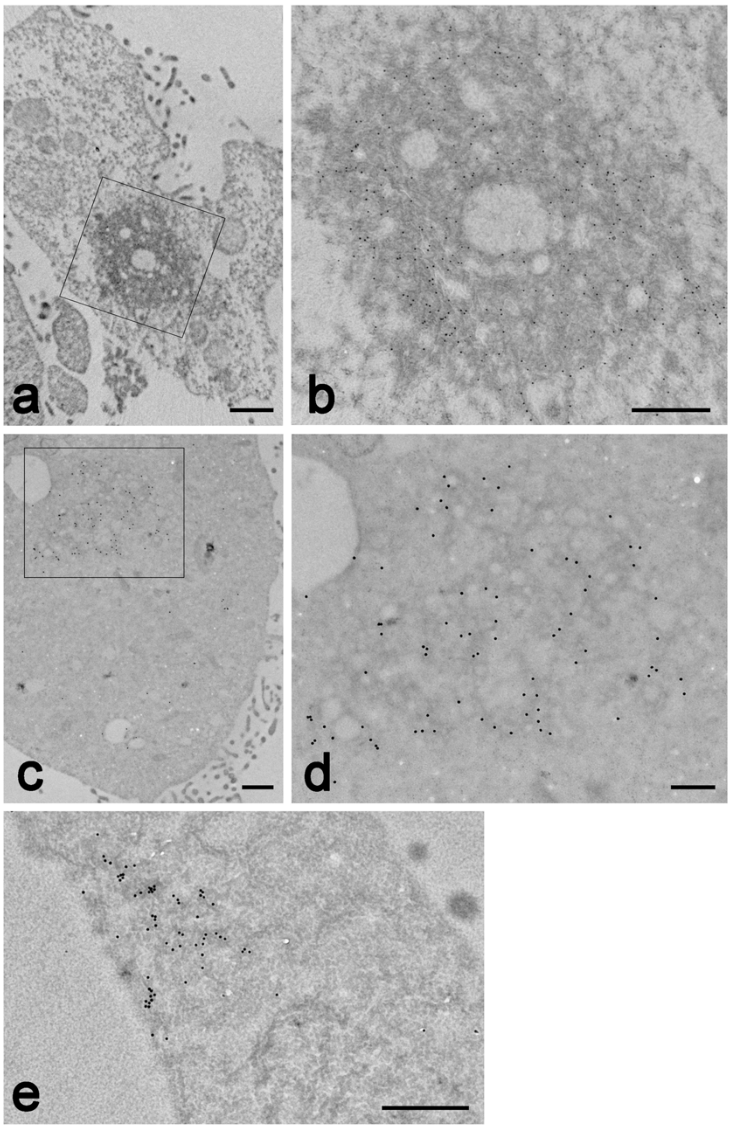

Supplementary Fig. 7. JRAB/MICAL-L2 and Rab8 are present at high concentrations in confined areas in the cytosol, related to Fig. 5. Immunoelectron microscopy of HeLa cells co-expressing GFP-JRAB/MICAL-L2 and HA-Rab8ADA.

Cells were immunostained for GFP (JRAB) (**a**–**d**) or HA (Rab8ADA) (**e**), and labeled with 10 nm protein A gold (**a**, **b**, and **e**), or 20 nm IgG gold (**c** and **d**). Boxed areas in **a** and **c** are enlarged in **b** and **d,** respectively. In some cases, sections were post-stained with uranyl acetate and lead citrate to enhance the contrast (**a**, **b,** and **e**). Note that the border between the immunoreactive area and surrounding cytoplasm is not membrane-bound. Scale bar, 1 μm (**a** and **c**), 500 nm (**b**, **d**, and **e**).

Supplementary Fig.8

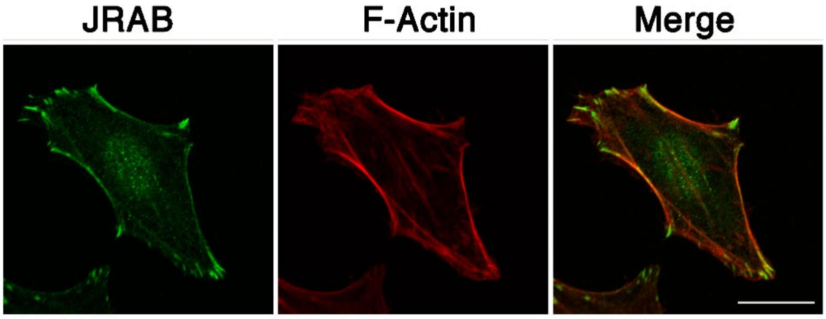

Supplementary Fig. 8. Liquid-like droplets were not observed in HeLa cells without exogenous JRAB/MICAL-L2, related to Fig. 5. HeLa cells were fixed and double-stained with an anti-JRAB/MICAL-L2 antibody (green) and rhodamine-phalloidin (red). Scale bar, 20 μm. More than 40 cells from four individual preparations were examined, and representative images are shown.

Supplementary Fig.9

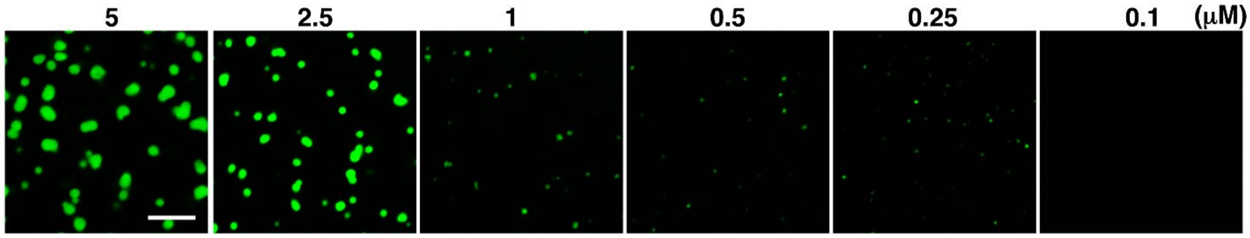

Supplementary Fig. 9. Characterization of the *in vitro* condensates of His-GFP-JRAB/MICAL-L2 and His-mCherry-Rab8ADA, related to Fig. 5i. Solutions containing His-GFP-JRAB (green) and His-mCherry-Rab8ADA at the indicated concentrations were prepared. Thirty minutes after the addition of crowding agent (5% PEG), each image was acquired. Scale bar, 10 μm. Experiments were repeated independently three times, with similar results.

Supplementary Fig.10

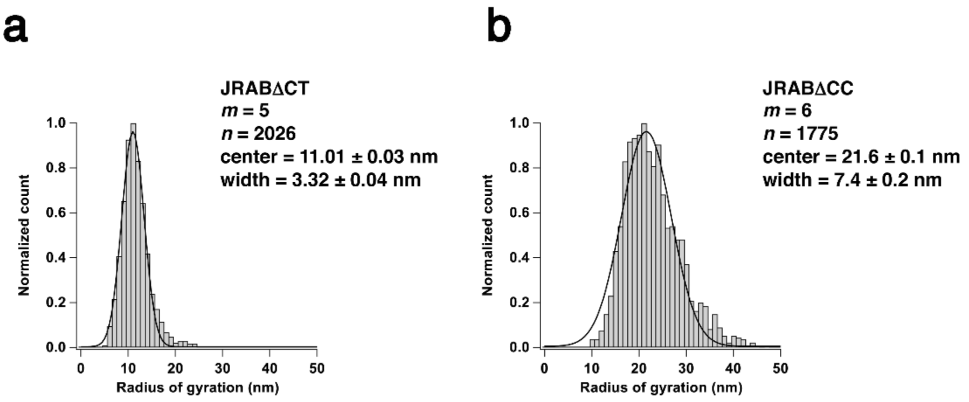

Supplementary Fig. 10. Histograms of the radius of gyration (R_g) for (a) JRABΔCT and (b) JRABΔCC, related to Fig. 6. The histograms were created with five molecules (*n* = 2,026) for JRABΔCT and six molecules (*n* = 1,775) for JRABΔCC, and normalized against the peak values. The solid line on the histogram corresponds to the fitted curve with Gaussian distribution.

Supplementary Fig.11

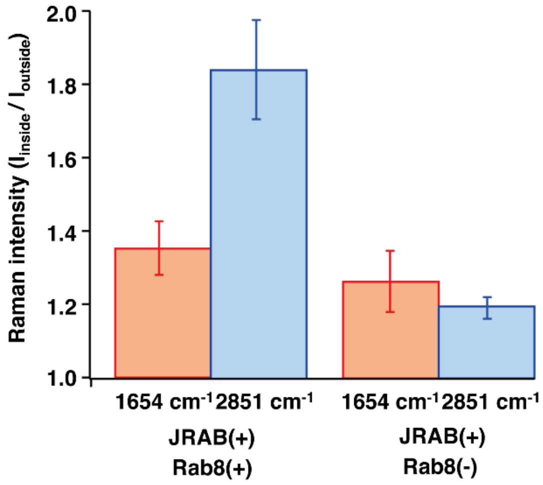

Supplementary Fig. 11. Raman intensity ratios between the interior and exterior of the droplets of GFP-JRAB/MICAL-L2 along with and without Rab8A, related to Fig. 5d–f. The Raman intensity ratios at 1,654 cm^{-1} and 2,851 cm^{-1} correspond to concentration of proteins and lipids, respectively, in the droplets. The error bars were created from the standard deviation of Raman spectral measurements taken from more than three different droplets. Source data are available in Supplementary Data 1.

Supplementary Fig.12

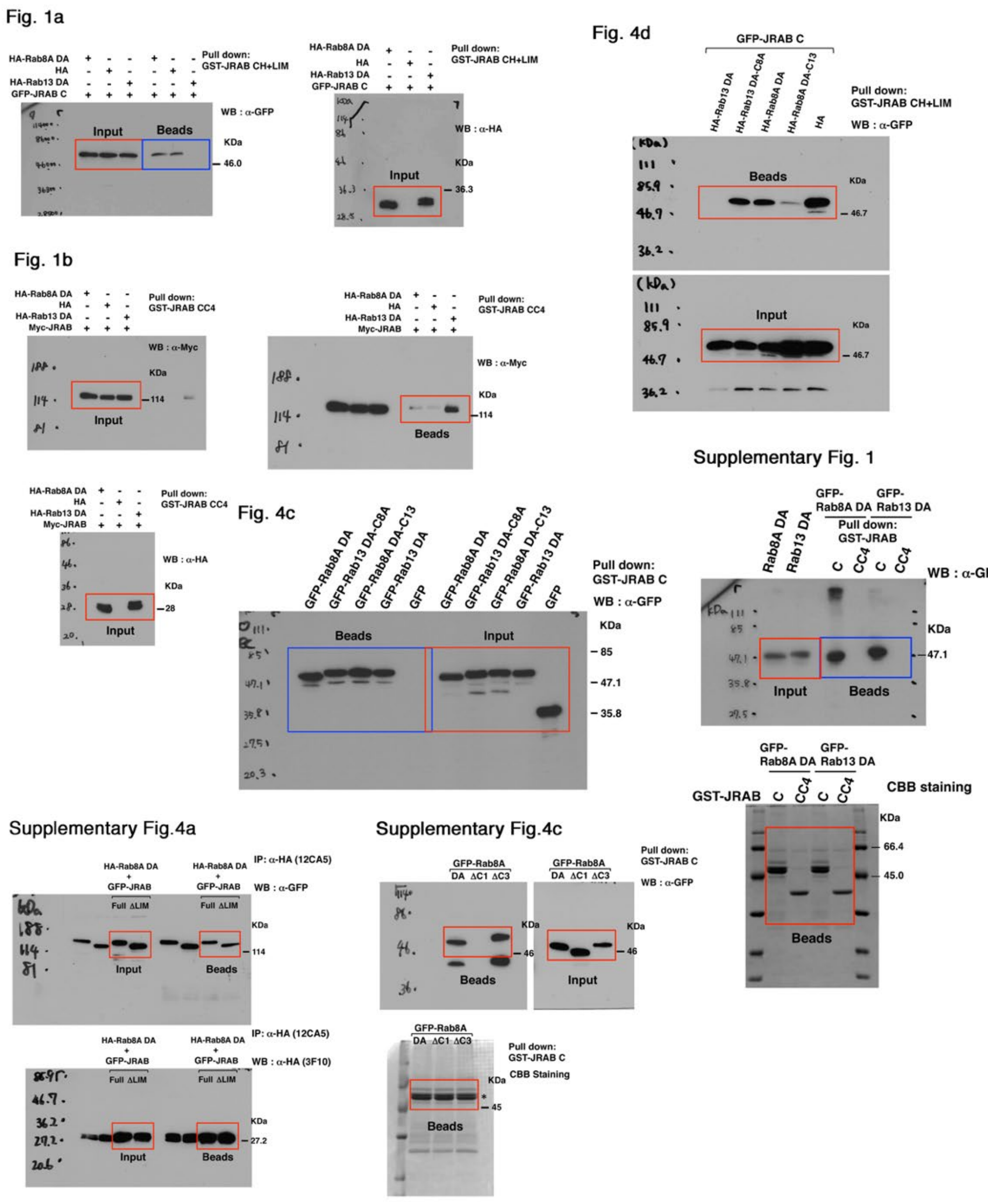

Supplementary Fig. 12. Full-length images of blots and gels. Uncropped images of scanned blots or gels in Fig. 1a, 1b, 4c, 4d, Supplementary Fig. 1, 4a and 4c. The red or blue square indicates each cropped area.

Supplementary Table 1. Resources used in this study.

REAGENT or RESOURCE	SOURCE	IDENTIFIER
Antibodies		
Mouse anti-hemagglutinin (HA) antibody (clone 12CA5)	Merck	Cat# 11583816001
Rat anti-HA antibody (clone 3F10)	Roche	Cat# 11867 423 001
Rabbit polyclonal anti-HA antibody	Santa Cruz Biotechnology	Cat# sc-805
Rabbit anti-GFP antibody	Thermo Fisher Scientific	Cat# A6455
Rabbit polyclonal anti-GFP(FL) antibody	Santa Cruz Biotechnology	Cat# sc-8334
Rabbit anti-Rab11 antibody	Zymed Laboratories Inc.	Cat# 71-5300
Anti-Calnexin antibody (clone AF18)	abcam	Cat# ab31290
Anti-Bip antibody	Stressgen	Cat# SPA-826
Mouse anti-Human Transferrin receptor antibody	Zymed Laboratories Inc.	Cat# 13-6800
Anti-human-CD147 antibody	BioLegend	Cat# 306202
Rat anti-MICAL-L1 antibody	Our lab	N/A
Rabbit anti-JRAB/MICAL-L2 antibody	Sakane et al. 2012	N/A
Peroxidase-conjugated AffiniPure Goat anti-Mouse lgG(H+L)	Jackson ImmunoResearch	Cat# 115-035-003
Peroxidase-conjugated AffiniPure Goat anti-Rabbit lgG(H+L)	Jackson ImmunoResearch	Cat# 111-035-003
Peroxidase-conjugated AffiniPure Goat anti-Rat lgG(H+L)	Jackson ImmunoResearch	Cat# 112-035-003
Cy3-conjugated AffiniPure Donkey anti-Rabbit lgG(H+L)	Jackson ImmunoResearch	Cat# 711-165-152
Alexa 647–conjugated Donkey anti-Mouse IgG antibody	Thermo Fisher Scientific	Cat# A31571
Goat anti–Rabbit IgG conjugated with 10 nm gold	BBI Solutions	Cat# EMGAR10
Goat anti–Rabbit IgG conjugated with 20 nm gold	BBI Solutions	Cat# EMGAR20
Protein A conjugated 10 nm colloidal gold	Homemade	Slot and Geuze,1985
Chemicals, Peptides, and Recombinant Proteins		
PEI-Max transfection reagent	Homemade	

Rhodamine–phalloidin	Thermo Fisher Scientific	Cat# R415
MitoTracker Red CMXRos	Thermo Fisher Scientific	Cat# M7512
Lipi-Red	DOJINDO	Cat# LD03
1,6-hexanediol	Sigma-Aldrich	Cat# 240117-50G
Brain PC	Avanti Polar Lipids	Cat# 840053P-25mg
Brain PS	Avanti Polar Lipids	Cat# 840032C-10mg
Recombinant DNA		
pEGFP-JRABwt	Sakane et al. 2010	N/A
pEGFP-JRABΔCT	Sakane et al. 2010	N/A
pEGFP-JRABΔLIM	Miyake et al., 2019	N/A
pEGFP-JRAB-C	Sakane et al., 2012	N/A
pEGFP-JRAB-IDR	Sakane et al., 2012	N/A
pEGFP-JRAB-IDR1	This paper	N/A
pEGFP-JRAB-IDR2	This paper	N/A
pEGFP-JRAB-IDR3	This paper	N/A
pEGFP-MICAL-L1	This paper	N/A
pcDNAHisMax-JRABΔCC	Sakane et al., 2016	N/A
pcDNAHisMax-JRABΔCT	Sakane et al., 2016	N/A
pGEX-6P-1-JRAB-CH+LIM	Sakane et al., 2012	N/A
pGEX-6P-1-JRAB-C	Sakane et al., 2012	N/A
pGEX-6P-1-JRAB-CC4	Sakane et al., 2016	N/A
pCIneoMyc-JRABwt	Terai et al. 2006	N/A
FRET-based JRAB indicator	Sakane et al. 2016	N/A
pEGFP-Rab8ADA	This paper	N/A
pEGFP-Rab8ADAΔC1	This paper	N/A
pEGFP-Rab8ADAΔC3	This paper	N/A
pEGFP-Rab13DA	Sakane et al., 2012	N/A
pEGFP-Rab8ADA-C13	This paper	N/A
pEGFP-Rab13DA-C8A	This paper	N/A
pmCherry-Rab8ADA	This paper	N/A
pCIneoHA-Rab8ADA	Yamamura et al, 2008	N/A
pCIneoHA-Rab8ADAΔC3	This paper	N/A

pCIneoHA-Rab8ADN	This paper	N/A
pCIneoHA-Rab13DA	Terai et al. 2006	N/A
pCIneoHA-Rab8ADA-C13	This paper	N/A
pCIneoHA-Rab13DA-C8A	This paper	N/A
pcDNAHisMax-GFP-JRAB	This paper	N/A
pcDNAHisMax-mCherry-Rab8ADA	This paper	N/A
Software and Algorithms		
R version 3.6.3	The R Project for Statistical Computing	https://www.r-project.org/
Igor Pro 8	WaveMatrics	https://www.wavemetrics.com/products/igorpro
ORIGIN8	Origin Lab	https://www.originlab.com/index.aspx?go=PRODUCTS/Origin
PSPredictor	Li et al. 2020	http://www.pkumdl.cn:8000/PSPredictor/
IUPred2A	Mészáros et al. 2018	https://iupred2a.elte.hu/
