# Supplementary material for: JRAB/MICAL-L2 undergoes liquid–liquid phase separation to form tubular recycling endosomes
Source: Commun Biol. 2021 May 11;4:551. doi: 10.1038/s42003-021-02080-7 (PMC8113518; doi:10.1038/s42003-021-02080-7)
Supplement: Supplementary file 2 — Description of Additional Supplementary Files [file 42003_2021_2080_MOESM2_ESM.pdf]

Description of Additional Supplementary Files

File Name: Supplementary Movie 1

Description: Time-lapse images of HeLa cells transfected with GFP-JRAB/MICAL-L2 and HA-Rab8ADA, related to Fig. 5c.

File Name: Supplementary Movie 2

Description: Time-lapse images of FRAP assay, related to Fig. 5h.

File Name: Supplementary Movie 3

Description: HS-AFM images of JRABΔCT (closed form) anchored on the Ni^{2+}/mica substrate through the N-terminal His6-tag, related to Fig. 6b.

File Name: Supplementary Movie 4

Description: HS-AFM images of JRABΔCC (open form) anchored on the Ni^{2+}/mica substrate through the N-terminal His6-tag, related to Fig. 6b.

File Name: Supplementary Movie 5

Description: Time-lapse series showing that droplets containing GFP-JRAB/MICAL-L2 and HA-Rab8ADA disappeared after the addition of 1,6-hexanediol (10%w/v), related to Fig. 7a

File Name: Supplementary Table 1

Description: Resources used in this study.
